# Ecological Restoration of Antibiotic-Disturbed Gastrointestinal Microbiota in Foregut and Hindgut of Cows

**DOI:** 10.3389/fcimb.2018.00079

**Published:** 2018-03-13

**Authors:** Shoukun Ji, Tao Jiang, Hui Yan, Chunyan Guo, Jingjing Liu, Huawei Su, Gibson M. Alugongo, Haitao Shi, Yajing Wang, Zhijun Cao, Shengli Li

**Affiliations:** ^1^State Key Laboratory of Animal Nutrition, Beijing Engineering Technology Research Center of Raw Milk Quality and Safety Control, College of Animal Science and Technology, China Agricultural University, Beijing, China; ^2^Laboratory of Animal Nutrition, College of Animal Science, Tarim University, Alar, China; ^3^Laboratory of Animal Nutrition, College of Animal Science and Technology, Shihezi University, Shihezi, China

**Keywords:** gut microbiota, antibiotics, microbial ecology, restoration, foregut, hindgut

## Abstract

Antibiotically disturbed gastrointestinal microbiota needs a long period time to be restored to normal, which may cause a series of problems to the host. The understanding of restoration of the biased microbiota by antibiotics remains largely unknown. Here, we investigated the microbiota shift in foregut (rumen) and hindgut (rectum) of lactating cows after antibiotics exposure as well as after antibiotics withdrawal with (Microbiota transplantation, MT group) or without (Control, CON group) microbiota transplantation. We were able to demonstrate that microbiota in both foregut and hindgut significantly changed after 3 or 14 days of antibiotics exposure, and the changes persisted over long period of time (>18 days) after withdrawing the antibiotics. We further observed a faster restoration of microbiota in both foregut and hindgut of MT group than CON group, microbiota in foregut was mainly benefited from microbiota transplantation by restoring the alpha-diversity as well as within-group similarity, while microbiota in hindgut was primarily benefited from microbiota transplantation by reestablishing the co-occurrence network (nodes number, edges number, density, modularity as well as closeness centrality). These results together expanded our understanding of restoration of the biased microbiota by antibiotics, and may also be instructive to deal with the delayed microbiota restoration at least in cows.

## Introduction

Man has experienced much beneficial impact from the widespread use of antibiotics for over half a century. The benefits include efficient killing of pathogens (Willing et al., 2011) and as growth promoters in animal husbandry (Cho et al., 2012). The demand and consumption of antibiotics is still growing rapidly worldwide (Laxminarayan et al., 2016). The adverse effects arising from the use of antibiotics such as, perturbed host resident microorganisms, increasing antibiotic-resistant pathogens, and direct negative effects on the host, has increased people' concern in recent years (Willing et al., 2011; Cho et al., 2012; Perez-Cobas et al., 2013; Morgun et al., 2015; Korpela et al., 2016; Langdon et al., 2016). Among these, dysbiosis of the microbiota that results from use of antibiotics has been associated with a large number of health problems as well as being implicated in modulation of the host metabolism, immune function and susceptibility to pathogens (Sekirov et al., 2008; Rooks et al., 2014; Boulangé et al., 2016; Langdon et al., 2016; Mahana et al., 2016).

It is well known that microbial fermentation in rumen and lower intestine supply most of the energy and protein required by ruminants (NRC, 2001; France and Kebreab, 2008). Maintaining a healthy gastrointestinal microbiota is critical for the health and productivity of ruminants. However, antibiotic administration in cows is widely adopted for treating infections such as metritis (Haimerl and Heuwieser, 2014) or mastitis (Vasquez et al., 2016), and preventing further infections (Scherpenzeel et al., 2014; Golder et al., 2016). Each cow might receive frequent antibiotic therapy during her lifetime because of the high rate of infections in both young calves (Walker et al., 2012) and adult cows (Pinedo et al., 2010), these may disturbed the indigenous microbiota as well as increased antibiotic-resistant genes in dairy cows (Wichmann et al., 2014; Chambers et al., 2015; Liu et al., 2016).

A healthy microbial community is essential for the health of the host (McKenney and Pamer, 2015). Ecological disturbances in the microbiota after antibiotic administration can persist for extended periods of time, and some taxa of indigenous bacteria might even not be recovered (Jernberg et al., 2010; Manichanh et al., 2010; Nobel et al., 2015; Korpela et al., 2016). The difficulties in restoration of indigenous bacterial community may go beyond our imagination as the disruption of microbiota by antibiotics has also accumulated over generations (Arnal et al., 2015; Blaser, 2016). Recently, there has been an increased interest in research on strategies that can be used to restore antibiotics disturbed gastrointestinal microbial ecosystem to normal.

Microbiota transplantation has been demonstrated as an efficient approach to reprogram gut microbiota in a critically disturbed microbial ecosystem in recurrent *Clostridium difficile* infection (Cammarota et al., 2014; Fuentes et al., 2014; Khoruts and Sadowsky, 2016; Li et al., 2016), and thus been recommended as a therapeutic method (Bagdasarian et al., 2015). Previous studies also demonstrated that rumen microbiota transplantation was effective to intervene in the metabolic disorders with diet-induced milk fat depression (Rico et al., 2014) or surgical correction of left-sided displacement of the abomasum (Rager et al., 2004). However, foregut (rumen) and hindgut (rectum) of cows harbored distinct microbiota (Godoy-Vitorino et al., 2012; Wang et al., 2017), and these raised two questions: (1) will microbiota transplantation be efficient in restoring the antibiotics disturbed gastrointestinal microbial ecology, and (2) will the response of microbiota in foregut and hindgut to the microbiota transplantation be different. To address these questions, we used lactating cows as animal model to characterize the microbial ecology in foregut and hindgut, analyzed the correlation of microbiota in foregut and hindgut, monitored microbiota change after administration of antibiotics, and compared the shift rate of microbiota diversity and co-occurrence network feature after antibiotics withdrawal with or without microbiota transplantation in foregut and hindgut, respectively.

## Materials and methods

### Animals

Fifteen ruminally fistulated lactating Holstein cows in their middle lactation stage (150 ± 10 days in lactation) were prepared 2 months before experiments, and were housed in a free stall pen at the Zhongdi Dairy Research Center (Beijing, China). The research center has been equipped with RIC® System (Roughage Intake Control System, Insentec B.V., Marknesse, Netherlands) and Heatime® Pro System (SCR Engineers Ltd., Netanya, Israel) to monitor the feed intake and activity, respectively, to verify cows in healthy condition during the experiment. All cows were fed a total mixed ration diet *ad libitum* containing 33% roughage and 67% concentrates (Supplementary Table S1) and had free access to clean water. All animal studies were approved by the Ethical Committee of the College of Animal Science and Technology (Project number 2016–2010) of China Agricultural University. Animal care and use were approved by complied with the Regulations for the Administration of Affairs Concerning Experimental Animals, National Committee of Science and Technology of China (14 November 1988) and Instructive Notions with Respect to Caring for Laboratory Animals, Ministry of Science and Technology of China (30 September 2006).

### Group assignment

Cows were randomly assigned to 3 groups with 5 cows in each group and marked with ear tags. Two groups were allocated to experimental groups and another group as donor group. After a 14-days adaptation period to the experimental conditions, rumen and rectum samples were collected from experimental cows.

### Antibiotics treatment

Ten cows from experimental groups received antibiotics by intramuscular injection for 14 days, which was one of most used antibiotic therapeutic strategy for ruminants in practice. The antibiotics consisted of penicillin (4.8 g per animal) and streptomycin (5.0 g per animal) at recommended dose, and each cow received antibiotics two times each day at 08:00 h and 20:00 h with 12 h a circle. Rumen and rectum samples were collected after 3 and 14 days of antibiotic administration.

### Microbiota transplantation

Microbiota transplantation approach was modified from previous protocol (DePeters and George, 2014). Briefly, rumen fluid was collected from donor cows and mixed evenly before transplantation to keep each experimental cow receiving the same microbiota. After antibiotic treatment, cows from one experimental group received rumen microbiota transplantation by transferring 10 L donor rumen fluid via rumen fistula (MT group), while cows from another experimental group received 10 L distilled deionized water (CON group). Both rumen fluid transplantation and water infusion were administrated once per day at 07:00 h before morning feeding for 3 continuous days. Rumen and rectum samples were collected at 4, 11, and 18 days after withdrawing antibiotics.

### Sample collection

Original rumen digesta of each cow was collected before morning feeding via rumen fistulas from the middle part of the ventral sac. The rumen fluids as foregut samples were obtained by squeezing the original digesta through four layers of sterile cheesecloth. Fecal digesta as hindgut samples were collected from rectum before morning feeding. All samples were stored in sterile tube and snap-frozen in liquid nitrogen immediately and then stored at −80°C until DNA extraction.

### DNA extraction and high throughput sequencing

Genomic DNA of rumen fluid and rectum digesta was extracted using a Qiagen DNA Extraction kit™ (Qiagen 51504, Hilden, Germany) following the manufacturer's protocol. Then 16S rRNA genes were amplified using barcoded primers targeting the V3-V4 region (Brown et al., 2016). Sequencing libraries were generated using the NEB Next Ultra DNA Sample Preparation kit (NEB, MA, USA) following the standard Illumina sample-preparation protocol (Caporaso et al., 2012) and then sequenced on an Illumina MiSeq platform (San Diego, CA, USA).

### Data processing

Sequence analyses were performed using QIIME pipeline (version 1.5.0) (Caporaso et al., 2011) as previously described (Ji S. et al., 2017). Quality control of the raw data was performed by FastQC (version 0.11.3). Paired-end reads from the original DNA fragments were merged using FLASH (version 1.2.7) (Magoc and Salzberg, 2011), and reads with ~420 bp were generated. Concatenated sequences were detected using USEARCH (version 6.1) and subsequently filtered out. Generated sequences were distributed into different samples based on barcodes, and the OTUs were defined by clustering sequences together with a 97% identity cut-off using UCLUST software (version 9.1) (Edgar, 2010) after removing the singletons and barcodes. Consequently, we identified 5,929,820 raw sequences (49,415 ± 11,597 sequences per sample) and 5,698,058 clean sequences (47,484 ± 11,114 sequences per sample). The RDP 11.5 database was used for taxonomic classification of generated OTUs. Rarefaction curves of detected OTUs or Shannon index in both foregut and hindgut demonstrated a high sequencing depth in current analysis (Supplementary Figure S1). 16S rRNA gene sequencing reads were deposited in the Genome Sequence Archive (http://gsa.big.ac.cn) in the BIG Data Center under accession numbers PRJCA000455.

### High-confidence OTUs

High-confidence OTUs were identified following a modified criteria (Sonnenburg et al., 2016). Briefly, in foregut and hindgut of pre-treated cows separately, OTUs with mean abundance higher than 0.001 were kept, then sub OTUs tables were recalculated and OTUs abundance higher than 0.01 were considered dominant OTUs while those lower than 0.001 were filtered out, only OTUs presented in more than 80% samples were considered as high-confidence OTUs.

### Network construction and topological feature analysis

To reduce rare OTUs in the data set, we used the high-confidence OTUs to construct the network. High-confidence OTUs in each group (pre-treated cows, antibiotics exposure cows and antibiotics withdrawal cows) were identified as described above. The OTUs table was generated by combining high-confidence OTUs in each group, the co-occurrence network was inferred based on the Spearman correlation matrix with *igraph* packages (1.0.1) in R software (Version 3.3.1), and the cut-off of false discovery rate (FDR) adjusted *P*-value of correlations was 0.001(Ma et al., 2016). Co-occurrence network in each group was inferred as sub-graph based on the high-confidence OTUs identified.

The nodes in networks represented OTUs, and the edges that connect these nodes represented correlations between OTUs. The node size represented the degree of node (the number of adjacent edges), the node color represented the vulnerability of node (the importance of a node in maintaining the connectivity of network). To calculate the vulnerability of each node in a network, we firstly measured the global efficiency (GE) which described the connectivity of a network between two random nodes (Latora and Marchiori, 2001), and then we removed nodes one by one to assess the GE change by equation: node vulnerability(i) = (GE-GE(i))/GE, the influence of a node on the network global efficiency reflected the vulnerability or importance of one node in a network. The edge color represented negative (red) or positive (blue) correlation of two connected nodes.

Topological features of each network were calculated with the *igraph* package (1.0.1) in R software (3.3.1). Parameters of node number, edge number, degree centrality, closeness centrality, betweenness centrality, vulnerability and modularity were calculated to describe a network.

### Statistical analysis

Alpha diversity indices were calculated using QIIME pipeline (version 1.5.0) (Caporaso et al., 2011), and the diversity (Shannon index), richness (Observed OTUs) and evenness (Pielou's evenness index) were calculated as previously described (Fuentes et al., 2014). The beta diversity indices, principal coordinate analysis (PCoA) and ANOSIM analysis between samples were determined based on Bray-Curtis metrics with *Vegan* package (Version 1.8-8) in R software (Version 3.3.1), to visually demonstrate the change path of microbiota, representative microbiota of each group was also calculated by the relative mean abundance and superimposed to the first two dimension of PCoA. Hypergeometric test in comparing the foregut and hindgut high-confidence taxa was performed with *VennDiagram* package (Version 1.6.17) in R (Version 3.3.1). Comparisons between groups were performed using a Wilcoxon test or Kruskal-Wallis test with R software (version 3.3.1). All data were presented as mean ± s.d., with ^*^P < 0.1, ^**^P < 0.05, ^***^P < 0.01.

## Results

### Characterization of gastrointestinal microbiota in lactating cows

**Figure 1:**
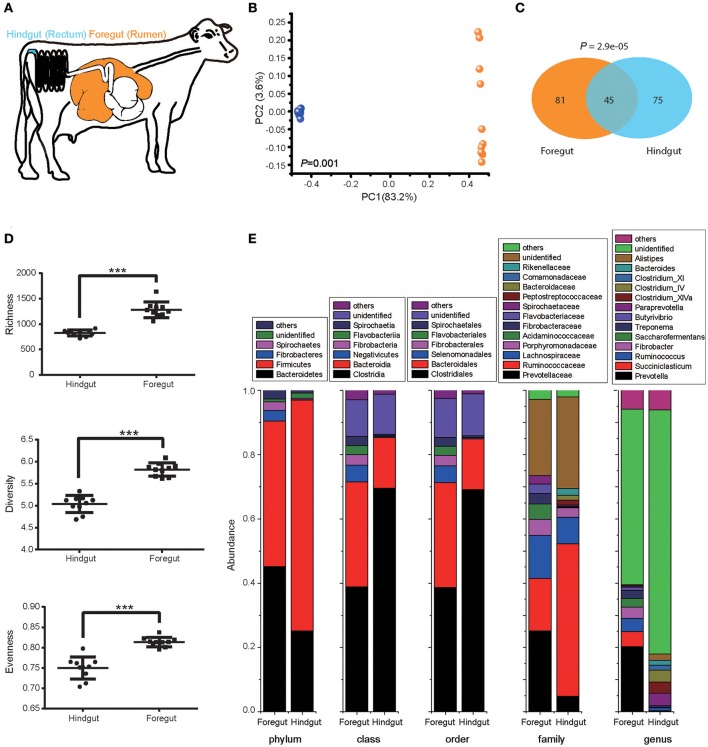
Foregut (rumen) and hindgut (rectum) harbored distinct microbiota. **(A)** Location of foregut and hindgut in gastrointestinal tract of lactating cow. **(B)** The microbiota in foregut and hindgut. Plot was drawn on the first two dimension of PCoA based on Bray-Curtis distance; statistical comparison of foregut and hindgut microbiota was performed with ANOSIM analysis. **(C)** High-confidence OTUs distribution in foregut and hindgut. Venn diagram showed the shared and unique high-confidence OTUs in foregut and hindgut, statistical comparison was performed with hypergeometric test. **(D)** Richness, diversity and evenness of microbiota in foregut and hindgut. **(E)** Microbiota community in foregut and hindgut at different levels of taxonomy; only taxa with abundance higher than 1% were shown. Data are expressed as mean ± s.d. ^***^P < 0.01.

We first explored the foregut (rumen) and hindgut (rectum) microbiota structure with 10 pre-treated cows under the normal animal husbandry conditions (Figure 1A). Foregut and hindgut harbored distinct microbial communities (Figure 1B; ANOSIM *P* < 0.05), and the composition of microbiota in foregut and hindgut differed in high-confidence OTUs (Figure 1C; *P* < 0.05), with higher richness, diversity and evenness in foregut (Figure 1D; *P* < 0.05). Though both foregut and hindgut were dominated by Bacteroidetes and Firmicutes at phylum level (abundance higher than 1%), Bacteroidetes in foregut were higher than those in hindgut (*P* < 0.05) while Firmicutes in foregut were lower than those in hindgut (*P* < 0.05). Additionally Fibrobacteres and Spirochaetes in foregut were also the dominant taxa and were higher compared to that in hindgut (*P* < 0.05). Compositional differences were also observed at the lower taxonomic levels (Figure 1E).

**Figure 2:**
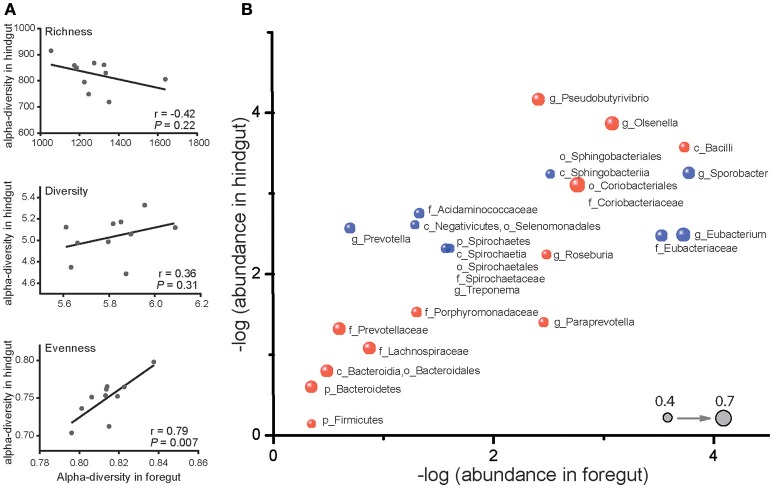
Correlation of microbiota in foregut and hindgut. **(A)** Richness, diversity and evenness correlations between foregut and hindgut microbiota. **(B)** Correlations of bacterial taxa in foregut and hindgut. Horizontal axis and vertical axis represent the mean value of relative bacterial abundance in foregut and hindgut, respectively. Bubbles in blue represent the positive correlations and those in red represent the negative correlations; bubble size represented the correlation coefficient (*r*); only bacterial taxa with correlation coefficient (*r*) higher than 0.4 were shown. Bacteria at phylum (p), class (c), order (o), family (f), and genus (g) level were included.

Despite the differences in microbiota of foregut and hindgut, we found out that evenness (*P* < 0.05) and diversity (*P* = 0.31) were positively correlated while richness (*P* = 0.22) was negatively correlated between foregut and hindgut microbiota (Figure 2A). We further observed 28 taxa at different taxonomic levels of microbiota to be remarkably correlated (Figure 2B; *P* < 0.05) with 14 taxa positively correlated (*r* > 0.40) and 14 taxa negatively correlated (*r* < −0.40). These data revealed that there was interaction between foregut and hindgut microbiota communities and indicated that the changes in foregut microbiota communities may affect lower gastrointestinal microbiota.

### Gastrointestinal microbiota change after antibiotics exposure

**Figure 3:**
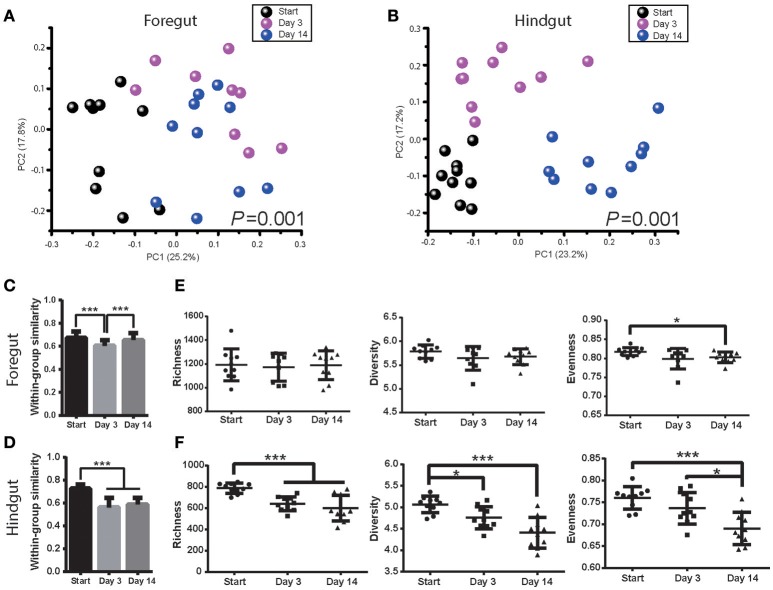
Antibiotics altered foregut and hindgut microbiota. **(A,B)** Microbiota changes in foregut **(A)** and hindgut **(B)** after antibiotics exposure. Plot was drawn on the first two dimension of PCoA based on Bray-Curtis distance; statistical comparison of microbiota was performed with ANOSIM analysis. **(C,D)** Within-group similarities of microbiota change in foregut **(C)** and hindgut **(D)** after antibiotics exposure. Within-group similarity was calculated from Bray-Curtis metrics as 1-(Bray-Curtis distance). **(E,F)** Richness, diversity and evenness change of microbiota in foregut **(E)** and hindgut **(F)** after antibiotics exposure. Data are expressed as mean ± s.d. ^*^P < 0.1, ^***^P < 0.01.

Foregut and hindgut microbiota were monitored after 3 and 14 days post antibiotics usage. Antibiotics had a marked effect on both foregut and hindgut microbiota communities (Figures 3A,B; ANOSIM *P* < 0.05). The within-group similarity of foregut microbiota decreased 3 days post antibiotics usage (Figure 3C; *P* < 0.05), while that of hindgut microbiota decreased after both 3 days and 14 days post antibiotics usage (Figure 3D; *P* < 0.05). Foregut microbiota richness and diversity were not affected by antibiotics however evenness tended to decrease after 14 days of antibiotics usage (Figure 3E; *P* < 0.1). Hindgut microbiota richness decreased at 3 days (*P* < 0.05), while richness, diversity and evenness decreased after 14 days of antibiotics usage (Figure 3F; *P* < 0.05).

**Figure 4:**
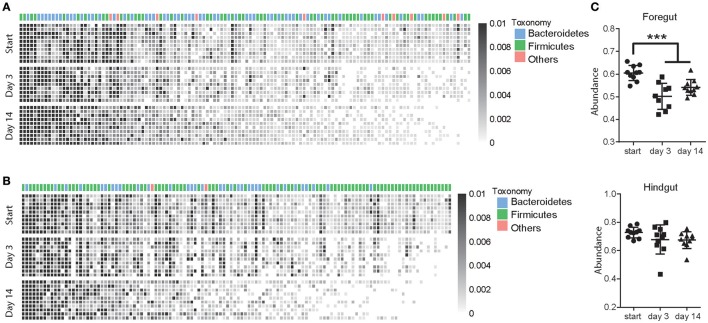
Antibiotics altered high-confidence OTUs in foregut and hindgut. **(A,B)** High-confidence OTUs change in foregut **(A)** and hindgut **(B)** after antibiotics usage. Each column represented one OTU and each row represented one individual cow from pre-treated period (Start) to 3 and 14 days after antibiotics exposure, taxonomic assignment is indicated at the top of each column. **(C)** Total abundance of high-confidence OTUs change in foregut and hindgut after antibiotics exposure. Data are expressed as mean ± s.d. ^***^P < 0.01.

A total of 126 OTUs in foregut and 120 OTUs in hindgut were identified as high-confidence OTUs using a cluster-free filtering approach as the description in method. Most of the high-confidence taxa were from phylum Bacteroidetes and Firmicutes in both foregut (Figure 4A) and hindgut (Figure 4B). These high-confidence taxa totally accounted for 60.4% in foregut and 72.8% in hindgut of total taxa abundance in pre-treated cows, and although they decreased in foregut (*P* < 0.05), that in hindgut was not affected after antibiotic usage (Figure 4C). In foregut, 45 high-confidence taxa decreased and 11 taxa increased in abundance after 3 days of antibiotics usage, while 43 taxa decreased and 12 taxa increased after 14 days of antibiotics usage (*P* < 0.05; Supplementary Table S2). In hindgut, 48 taxa decreased and 7 taxa increased after 3 days of antibiotics usage, 53 taxa decreased and 6 taxa increased after 14 days of antibiotics usage (Supplementary Table S3; *P* < 0.05).

### Foregut microbiota change after microbiota transplantation

To perform the microbiota transplantation, we chose another 5 healthy fistulated cows as rumen microbiota donors, collected and mixed the rumen fluid from donor cows evenly before transplantation to keep each experimental cow receiving the same microbiota. The microbiota community in foregut of donor cows showed higher similarities with pre-treated cows compared to antibiotics treated cows in foregut (Supplementary Figure S2). However, microbiota composition difference between donor and pre-treated cows could also be detected at different taxonomic levels (Supplementary Figure S3).

**Figure 5:**
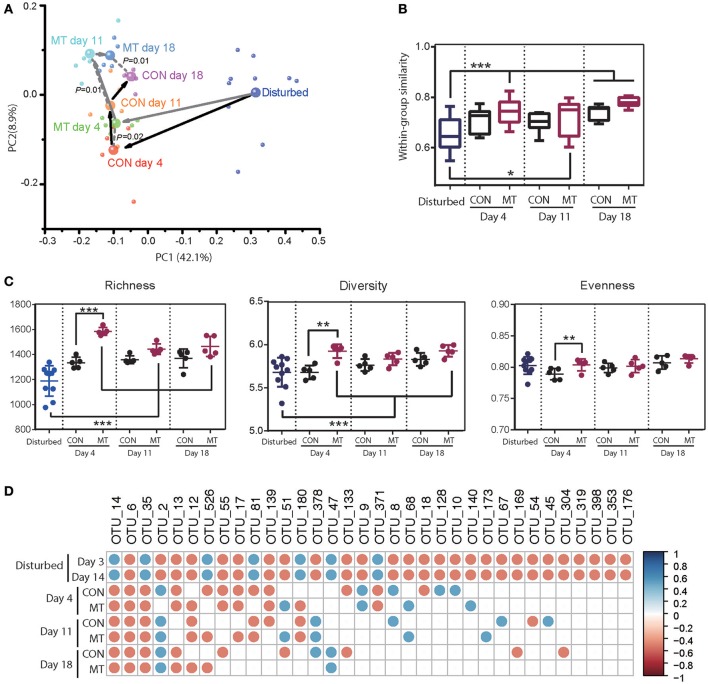
Restoration of foregut microbiota with or without microbiota transplantation. **(A)** Microbiota transplantation altered foregut microbiota community. Each small bubble represents one individual cow microbiota and big one represents the representative microbiota in each group. Arrows in dark represent the change in microbiota of CON group and those in gray represent the change in microbiota of MT group cows in the first two dimensions of PCoA. Microbiota from CON and MT group cows at same time point were compared with ANOSIM analysis. **(B)** Within-group similarities of foregut microbiota in CON and MT group changed over time. **(C)** Richness, diversity and evenness of foregut microbiota in CON and MT group changed over time. **(D)** The restoration of antibiotic disturbed high-confidence OTUs in foregut of CON and MT group over time. All OTUs were compared with those in pre-treated cows (Start group as shown in Figure 4); only significantly changed OTUs at both 3 and 14 days post antibiotics administration were shown; blue dots represent significant increase and red dots represent significant decrease. Data are expressed as mean ± s.d. ^*^P < 0.1, ^**^P < 0.05, ^***^P < 0.01.

Ten antibiotics pre-treated cows (antibiotics were firstly administered for 14 days) were randomly assigned to 2 groups with one group receiving microbiota transplantation (MT group) and another one receiving 10 L distilled deionized water (CON group), the foregut microbiota was monitored at 4, 11, and 18 days after antibiotics withdrawal. Foregut microbiota communities were indistinguishable in 2 groups at the end of antibiotics usage (Supplementary Figure S4), and changed immediately in both groups after antibiotics usage was stopped. However the foregut harbored distinct microbiota community in CON and MT group at 4, 11, and 18 days after withdrawing antibiotics usage (ANOSIM *P* < 0.05; Figure 5A). These observations suggested that microbiota transplantation may alter the foregut microbiota community during restoration of foregut microbiota from antibiotics disturbance.

Comparison of the microbiota communities in MT and CON groups revealed a higher shift rate after microbiota transplantation. In MT group, the within-group similarities increased at 4 and 18 days (*P* < 0.05) and tended to increase at 11 days (*P* < 0.1), however that in CON groups did not increase until 18 days after antibiotics withdrawal (*P* < 0.05; Figure 5B). Microbiota transplantation also had a marked effect on richness, diversity and evenness of foregut microbiota (Figure 5C). At 4 days after withdrawing antibiotics, diversity, richness and evenness of MT group were higher than those of CON group (*P* < 0.05). When comparing the foregut microbiota after antibiotics withdrawal to the disturbing microbiota community, richness in MT groups increased at day 4, 11, and 18 (*P* < 0.05), and diversity in MT group increased at day 4 and 18 (*P* < 0.05), however, the diversity, richness and evenness of CON group did not change at any time point during this experiment. Additionally, most of the changed high-confidence taxa in foregut restored in both CON and MT group, however at day 18 after antibiotics withdrawal, 12 taxa in CON group and 8 taxa in MT group differed from the pre-treated cows (Figure 5D).

### Hindgut microbiota change after microbiota transplantation

**Figure 6:**
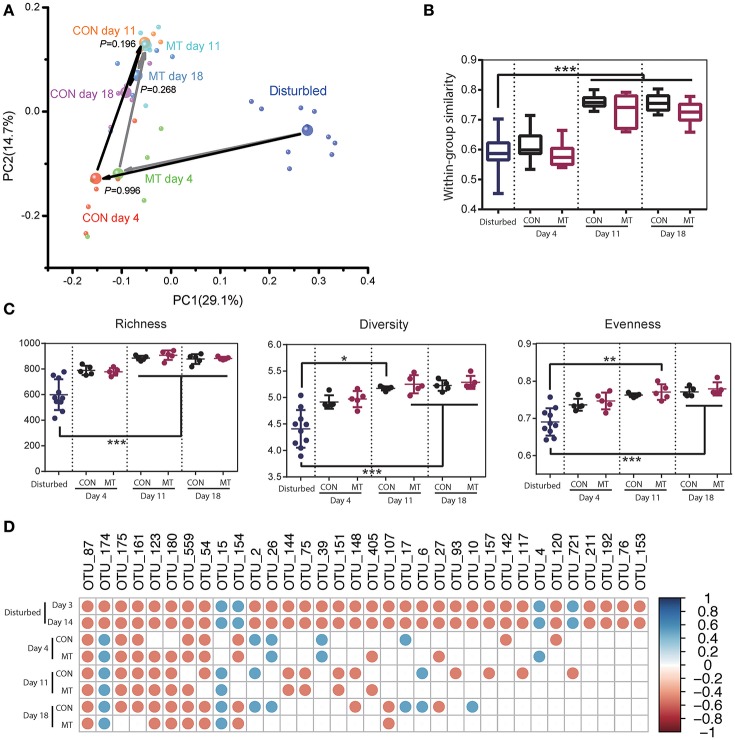
Restoration of hindgut microbiota with or without microbiota transplantation. **(A)** Hindgut microbiota change for CON and MT groups in the first two dimensions of PCoA. Each small bubble represents one individual cow microbiota and big one represents the representative microbiota in each group. Arrows in dark represent the microbiota change for CON group and those in gray represent the microbiota change for MT group. Microbiota from CON and MT groups at same time point were compared with ANOSIM analysis. **(B)** Within-group similarities of hindgut microbiota in CON and MT groups changed over time. **(C)** Richness, diversity and evenness of hindgut microbiota in CON and MT groups change over time. **(D)** The restoration of antibiotic disturbed high-confidence OTUs in hindgut of CON and MT groups over time. All OTUs compared with those in pre-treated cows (Start group as shown in Figure 4); only significantly changed OTUs at both 3 and 14 days post antibiotics administration were shown; blue dots represent significant increase and red dots represent significant decrease. Data are expressed as mean ± s.d. ^*^P < 0.1, ^**^P < 0.05, ^***^P < 0.01.

The hindgut microbiota of CON and MT group was also monitored at 4, 11, and 18 days after antibiotics usage withdrawal. Rapid changes were observed in both groups after antibiotics withdrawal, and microbiota communities were indistinguishable at all the time points (Supplementary Figure S5; Figure 6A). The within group similarities and richness did not increase until day 11 in both groups (Figures 6B,C; *P* < 0.05). However, the diversity and evenness increased at day 11 and day 18 for MT cows, and increased only at day 18 for CON cows (Figure 6C; *P* < 0.05). A more detailed analysis of the changes microbiota after microbiota transplantation in hindgut revealed that the high-confidence rebounded in a higher rate for MT cows than CON cows with 18 taxa in CON group and 9 taxa in MT group consistently differing from the pre-treated cows at day 18 after antibiotics withdrawal (Figure 6D). These data suggested that although microbiota in hindgut benefited less compared to the foregut after microbiota transplantation, microbiota transplantation may also affect the microbiota in the lower intestine.

### Network feature change in foregut and hindgut

**Figure 7:**
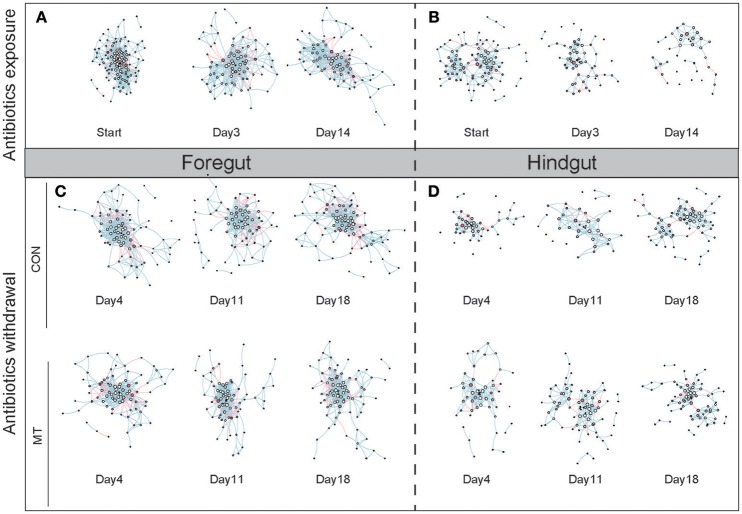
The co-occurrence network change in foregut and hindgut after antibiotics exposure and antibiotics withdrawal. **(A)** The co-occurrence network change in foregut after antibiotics exposure. **(B)** The co-occurrence network change in hindgut after antibiotics exposure. **(C)** The co-occurrence network change in foregut after antibiotics withdrawal. **(D)** The co-occurrence network change in hindgut after antibiotics withdrawal. The nodes represented high-confidence OTUs in each sub-data sets, the size of each node represented the node degree value, the color of each node represented the node vulnerability value. The edge stands for significant correlation between two nodes (*P* < 0.001), the edge color represented negative (red) or positive (blue) correlation of two connected nodes.

We inferred co-occurrence networks for foregut and hindgut of lactating cows, respectively, and created sub-graphs based on the high-confidence OTUs detected in each group (Figure 7). We firstly examined the structure change of networks during antibiotics exposure as well as after antibiotics withdrawal in foregut and hindgut, and observed that both nodes (Supplementary Figures S6a,b) and edges (Supplementary Figures S6c,d) number decreased after antibiotics exposure in foregut or hindgut. After antibiotics withdrawal, the number of nodes and edges in foregut maintained under low degree in MT group or CON group, however, the number of nodes and edges in hindgut rebounded, and a higher shift rate were observed in MT group than CON group (Supplementary Figures S6a–d). The density of a network is the ratio of the number of edges and the number of possible edges, we observed microbiota network density increased after antibiotics exposure, whereas after antibiotics withdrawal, MT group decreased faster than CON group in both foregut and hindgut (Supplementary Figures S6e,f). The modularity of a network with respect to some division (or vertex types) measures how good the division is, we found that MT group and CON group had similar modularity index change rate in foregut, while modularity index of MT group rebounded more fast than that of CON group after antibiotics withdrawal in hindgut (Supplementary Figures S6g,h). Taken together, microbiota transplantation promoted restoration of microbiota co-occurrence network in hindgut.

To further illustrate the network change during antibiotics exposure as well as after antibiotics withdrawal, we then measured the distribution of microbiota network degree, betweenness, closeness and vulnerability in foregut and hindgut, respectively. Degree and betweenness roughly followed a power-law distribution while closeness and vulnerability roughly followed a binomial distribution (Supplementary Figure S7), indicated scale-free networks we achieved and different OTUs in these networks may contribute differently in maintaining the network connectivity. Although we observed the distribution change of degree, betweenness, closeness and vulnerability in both foregut and hindgut in our monitored time-points (Supplementary Figure S7), closeness distribution was most sensitive to the antibiotics exposure, because only closeness distribution significantly differed after both short time (3 days) and long-time (14 days) antibiotics exposure in foregut and hindgut (Supplementary Figure S7; *P* < 0.05). Additionally, when we looked into the correlations of network degree, betweenness, closeness and vulnerability (Supplementary Figure S8), positive correlations were detected in each pairs of distribution parameters (*P* < 0.05), and vulnerability correlated with other three parameters with high correlation coefficient in both foregut (*r* > 0.48) and hindgut (*r* > 0.53). Thus, distribution of closeness and vulnerability was emphasized in assessing the dynamic change of network structure in current experiment.

Closeness indicated the distance of one node to others, and the closeness distribution reflected the tightness of network. We observed the closeness increased in both foregut and hindgut after antibiotics exposure (Supplementary Figure S7; *P* < 0.05). In foregut, closeness distribution restored only at day 11 in CON group after antibiotics withdrawal (*P* > 0.05), however increased again at day 18 (*P* < 0.05), and closeness distribution maintained under high level in MT group at all the monitored time-points (*P* < 0.05). In hindgut, closeness distribution in CON group maintained under high level at day 4 and day 11 after antibiotics withdrawal (*P* < 0.05), and decreased at day 18 with a still higher distribution than that of initial state (*P* < 0.05), the closeness distribution in MT group was higher than that of initial profile at day 4 after antibiotics withdrawal (*P* < 0.05), and restored to initial profile at day 11 and day 18 after antibiotics withdrawal (*P* > 0.05).

Most node vulnerability values distributed around zero in foregut and hindgut, meaning that these nodes had little influence on the network global efficiency (Supplementary Figure S7). However, some nodes with high vulnerability score were observed, indicating these nodes played important role in maintaining a network and acted as central nodes. We observed that the top 2 nodes with highest vulnerability in pre-treated cows were OTU49 (Clostridiales at Order level) and OTU493 (*Prevotella* at Genus level) in foregut and OTU99 (*Paraprevotella* at Genus level) and OTU205 (Bacteroidetes at Phylum level) in hindgut, the vulnerability values of these nodes in foregut shifted in similar rate in CON group and MT group after antibiotics exposure and antibiotics withdrawal, however, a more positive restoration was observed in MT group than that in CON group in hindgut (Supplementary Figure S9).

## Discussion

The gastrointestinal microbial ecosystem plays a variety of important roles in animal physiology and gut homeostasis (Clemente et al., 2012; Tremaroli and Bäckhed, 2012; Boulangé et al., 2016), and gastrointestinal microbial disorders have been demonstrated to be related to multiple host diseases (Fuentes et al., 2014; Marchesi et al., 2016; Zheng et al., 2016; Ishikawa et al., 2017). Antibiotics have benefited humans a lot by killing pathogens (Willing et al., 2011) and as growth promoters in animal husbandry (Cho et al., 2012), which also played important roles currently (Laxminarayan et al., 2016), however, the side effects of antibiotics exposure such as perturbing host resident microorganisms has raised people's concern (Isaac et al., 2016). Understanding the influence of antibiotics on gastrointestinal microbiota as well as the microbial restoration after antibiotics exposure is important to deal with the gastrointestinal microbial disorders caused by antibiotics. Ruminants have a unique digestive structure with rumen as foregut which harbors distinct microbiota from hindgut (Wang et al., 2017), which make it convenient to monitor the microbiota shift in foregut and hindgut, respectively. In this study we used lactating cows as animal model, treated cows with antibiotics to disturb the microbiota in foregut and hindgut, and illustrated the microbiota ecology shift after antibiotics withdrawal in foregut and hindgut, respectively.

Microbiota differences in the foregut and hindgut of lactating cows have been illustrated previously depending on distinct ecological environment, feed substrate and function (Godoy-Vitorino et al., 2012; Wang et al., 2017). Similar differences have also been observed in different gastrointestinal sites of the mice (Gu et al., 2013) and humans (Zhang et al., 2014). In this trial, the foregut harbored a much higher richness, diversity and evenness for microbiota than the hindgut, and our data also showed the correlation between microbiota in foregut and hindgut, which raised the question of how the microbiota in foregut affected that in hindgut in ruminants. Previous studies have demonstrated that intragastric infusion of xenomicrobiota may induce the xenomicrobiota colonization in the intestine (Fuentes et al., 2014; Li et al., 2016), while oral bacterial intake by animals or humans resulted in different intestinal microbiota (Lee et al., 2013; Petrof and Khoruts, 2014), which indicated that microbial importation into the gut might affect the microbiota in lower intestine. We observed that although the evenness and diversity of microbiota correlated positively, microbial richness correlated negatively between foregut and hindgut. Additionally, both negative and positive correlations between foregut and hindgut bacterial taxa could be observed, which hinted that the alteration of microbiota in foregut may also influence that in hindgut, and different bacterial taxa may regulate the microbiota in lower intestine with various efficiencies.

The influence of antibiotics on gastrointestinal microbiota has been widely explored in mice (Cho et al., 2012; Nobel et al., 2015) and humans (Isaac et al., 2016). Here we corroborated that either short (3 days) or long term (14 days) antibiotics exposure altered the microbiota in foregut and hindgut in lactating cows. The main function of antibiotics is to kill bacteria, however the antibiotic sensitivity of different bacteria differed (Morgun et al., 2015). This might explain why abundance of some bacteria taxa in foregut or hindgut increased. Commonly, antibiotics affected host in three modes: direct effects on the host, remaining antibiotic resistant microbes or depletion of microbiota (Morgun et al., 2015). Interestingly, here we confirmed that antibiotics may decrease the within-group similarity of microbiota, which reflected the decrease in maturity or stability of microbiota after antibiotics exposure (Jami et al., 2013). Based on the ecological niche theory, vacant niche facilitated the xenomicrobiota colonization, and these xenomicrobiota may include the pathogenic microorganism (Lee et al., 2013; Caballero et al., 2015). Thus, our findings here hinted that de-maturation of microbiota in gut may be another hazard factor of exposure to antibiotics.

A previous study showed that near total exchange of rumen microbiota in two cows disturbed the foregut microbiota, and the microbiota in both cows returned to their original profile after at least 14 days (Weimer et al., 2010), which suggested that the foregut microbiota may re-established after being challenged, meanwhile, the foregut microbiota restoration might last over a long time. Previous studies have also demonstrated that the altered microbiota by antibiotics were not easy to recover after antibiotics withdrawal, and thus could affect the host physiological homeostasis over a long period time (Jernberg et al., 2010; Manichanh et al., 2010; Nobel et al., 2015; Korpela et al., 2016), Although we monitored the foregut and hindgut microbiota change for 18 days after withdrawing the antibiotics, microbiota compositions in both segments did not return to their original profiles. These results supported the long term impact of antibiotics on microbiota in both foregut and hindgut, and a much longer monitoring period might be required before observing a fully restored microbiota.

It was a universal phenomenon that the restoration rate of microbiota after being disturbed by feed alteration, antibiotics usage or other gut disorders to be slower than expected, the host might suffer from low transition velocity of microbiota (Cho et al., 2012; Korpela et al., 2016; Sonnenburg et al., 2016). Thus quick restoration of the biased microbiota may be more crucial for maintaining host health and feed digestibility. Theoretically, supplying gut microbiota from healthier donor cows to antibiotics treated cows may introduce xenomicrobiota into recipients' gut to fill the vacant ecological niches, thus promoted restoration of the biased microbioata by antibiotics based on ecological niche theory, and this concept has been clinically applied in human as fecal microbiota transplantation (FMT) to adjust the gut microbiota disorders (Cammarota et al., 2014; Khoruts and Sadowsky, 2016; Li et al., 2016). Previous studies also observed in lactating cows that microbiota transplantation was effective to intervene in the metabolic disorders with diet-induced milk fat depression (Rico et al., 2014) or surgical correction of left-sided displacement of the abomasum (Rager et al., 2004). Our data here demonstrated that microbiota transplantation promoted restoration of the bias microbiota in dealing the delay of microbiota restoration.

Previous study has demonstrated that microbiota transplantation might promote the restoration of disturbed gut microbiota as well as the bacterial co-occurrence network (Fuentes et al., 2014). Interestingly, we observed that although microbiota in both foregut and hindgut benefited from microbiota transplantation, microbiota transplantation promoted restoration of the bacterial co-occurrence network mainly in hindgut. These new findings provided the basic information that microbiota in different gut segments might respond differently to the xenomicrobiota infusion, and which may be a universal phenomenon, as we have also observed different microbiota response in different gut segments in our previous study with mice model (Ji S. K. et al., 2017). In practice, it is not easy to infuse the microbiota to target intestinal segments such as cecum or colon, and intragastric infusion or oral intake were the most acceptable ways (Youngster et al., 2014; Borody et al., 2015), thus further understanding of the response of microbiota in different gut segments may be helpful to better regulate the gastrointestinal microbiota.

As demonstrated in this study, antibiotics disturbed both foregut and hindgut microbiota, and the restoration of microbiota needed a long period of time after antibiotics withdrawal. Adjusting the disturbed microbiota timely could benefit the host. Our data demonstrated that microbiota transplantation promoted the restoration of microbiota in both foregut and hindgut, revealing that microbiota transplantation could be an effective and practical measure in dealing with the delayed gut microbiota restoration. Additionally, foregut and hindgut harbored distinct microbial ecology, microbiota in foregut and hindgut might respond differently to the xenomicrobiota infusion. These suggested that gut segments should also be under consideration when we targeted to regulate the gut microbial ecology.

## Data accessibility

All data represented in this study have been deposited in the Genome Sequence Archive (http://gsa.big.ac.cn) in the BIG Data Center under accession numbers PRJCA000455.

## Author contributions

SL and SJ designed the study; HY, SJ, and TJ performed the experiments; TJ, CG, and JL collected and prepared samples for sequencing; HY performed sequencing and sequencing analysis with technical assistance from YW and SJ performed statistical interpretation and analyses with technical assistance from ZC, SJ, and HY took primary responsibility for writing the manuscript with English improved by HWS, HTS, and GA. All authors discussed the results and commented on the manuscript.

### Conflict of interest statement

The authors declare that the research was conducted in the absence of any commercial or financial relationships that could be construed as a potential conflict of interest. The reviewer MS and handling Editor declared their shared affiliation.
