# Supplementary material for: Ecological Restoration of Antibiotic-Disturbed Gastrointestinal Microbiota in Foregut and Hindgut of Cows
Source: Front Cell Infect Microbiol. 2018 Mar 13;8:79. doi: 10.3389/fcimb.2018.00079 (PMC5859144; doi:10.3389/fcimb.2018.00079)
Supplement: Supplementary file 1 [file Image1.pdf]

Supplementary Figures and legends (S1-8)

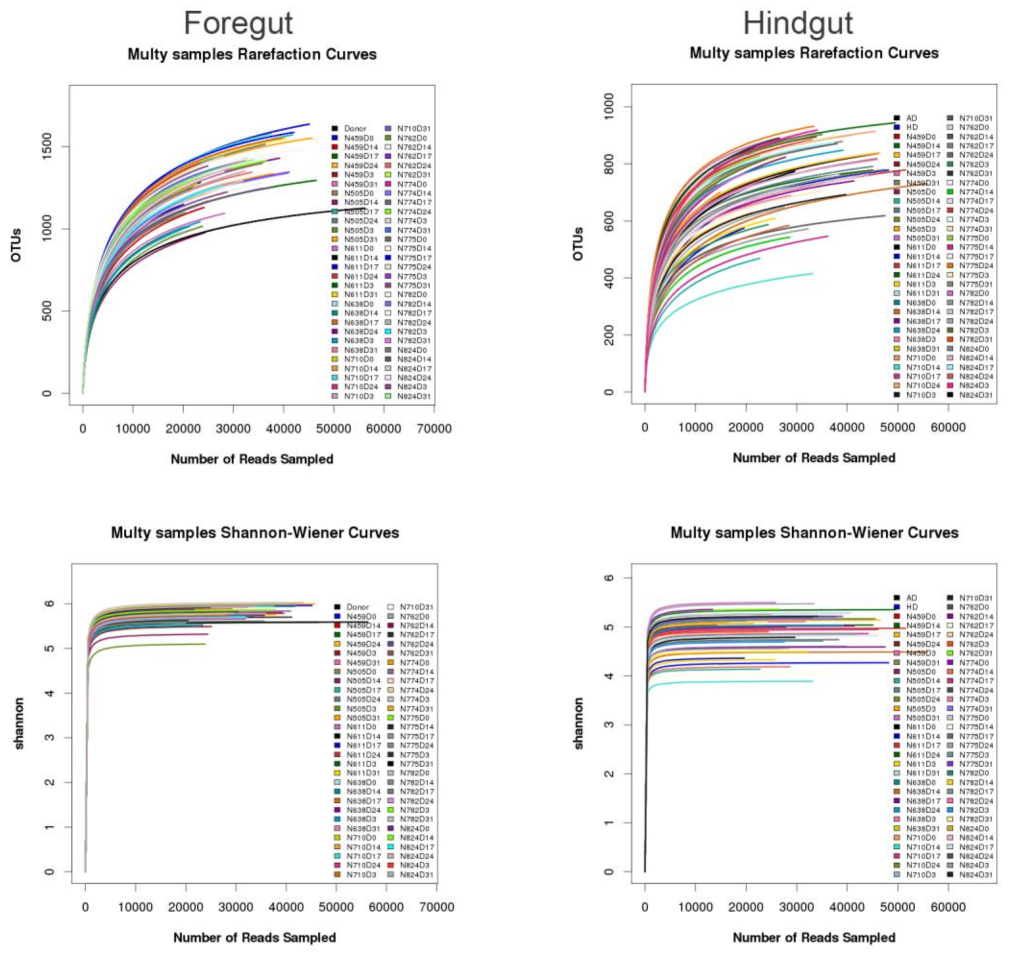

Supplementary Figure S1 Rarefaction curves of detected OTUs and Shannon index in foregut and hindgut.

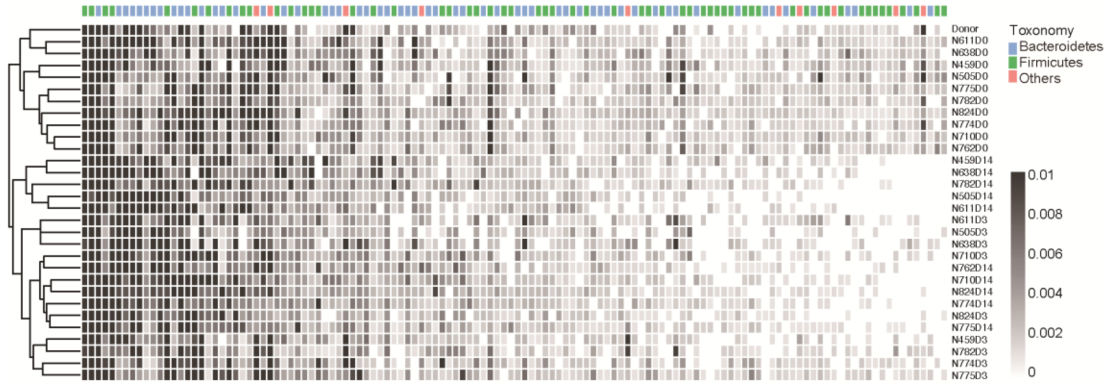

Supplementary Figure S2 High-confidence OTUs similarities of rumen microbiota in donor, pre-treated and antibiotics treated cows. Each column represented one OTU and each row represented one individual cow's microbiota from pre-treated period (D0) to 3 (D3) and 14 (D14) days post antibiotics administration; taxonomic assignment was indicated at the top of column. Cluster was calculated by row with Pearson correlation.

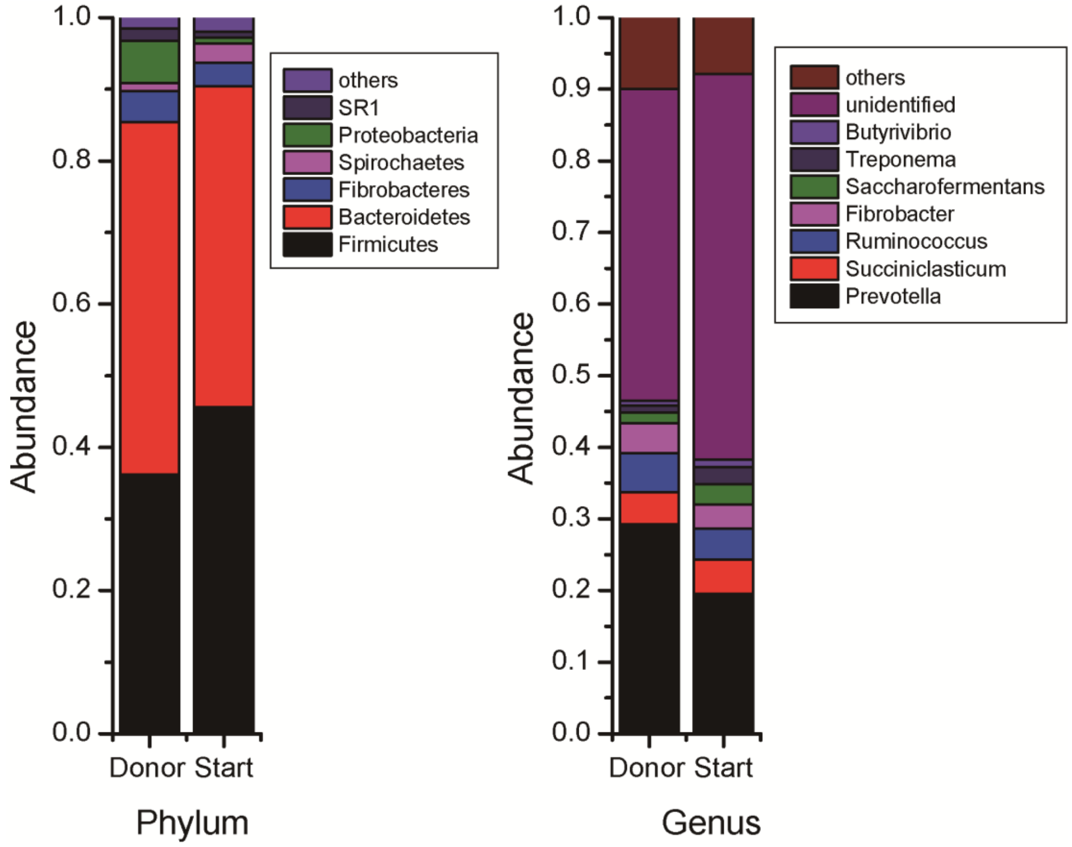

Supplementary Figure S3 Foregut microbiota composition of donor and pre-treated cows at phylum and genus level; only taxa with abundance higher than 1% were shown.

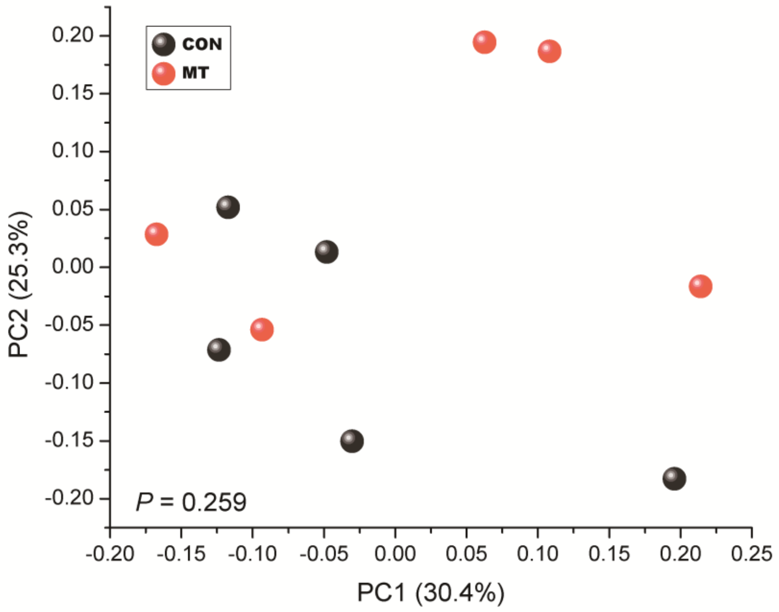

Supplementary Figure S4 Foregut microbiota similarities for CON and MT groups at the end of antibiotics administration (Day 14). Microbiota from CON and MT groups were compared with ANOSIM analysis.

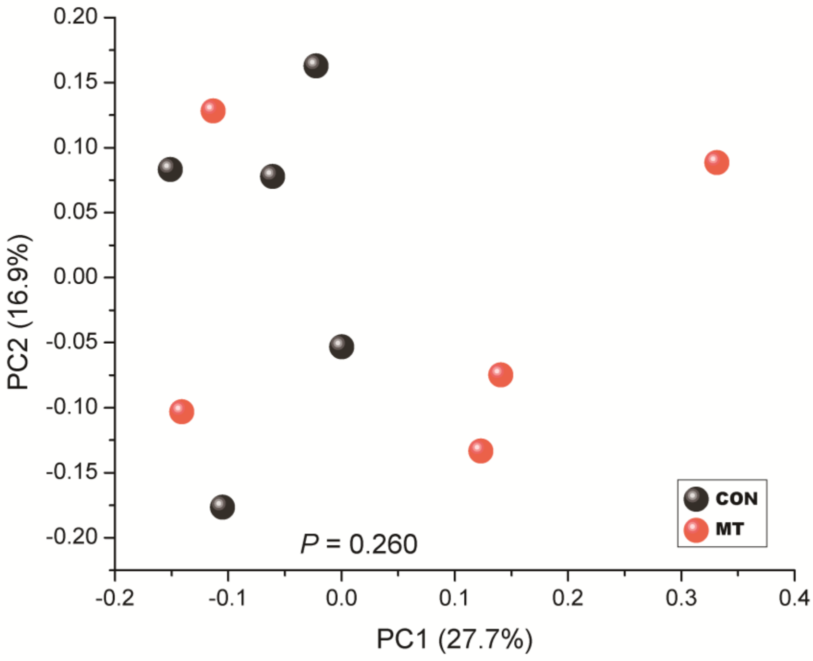

Supplementary Figure S5 Hindgut microbiota similarities for CON and MT groups at the end of antibiotics administration (Day 14). Microbiota from CON and MT groups were compared with ANOSIM analysis.

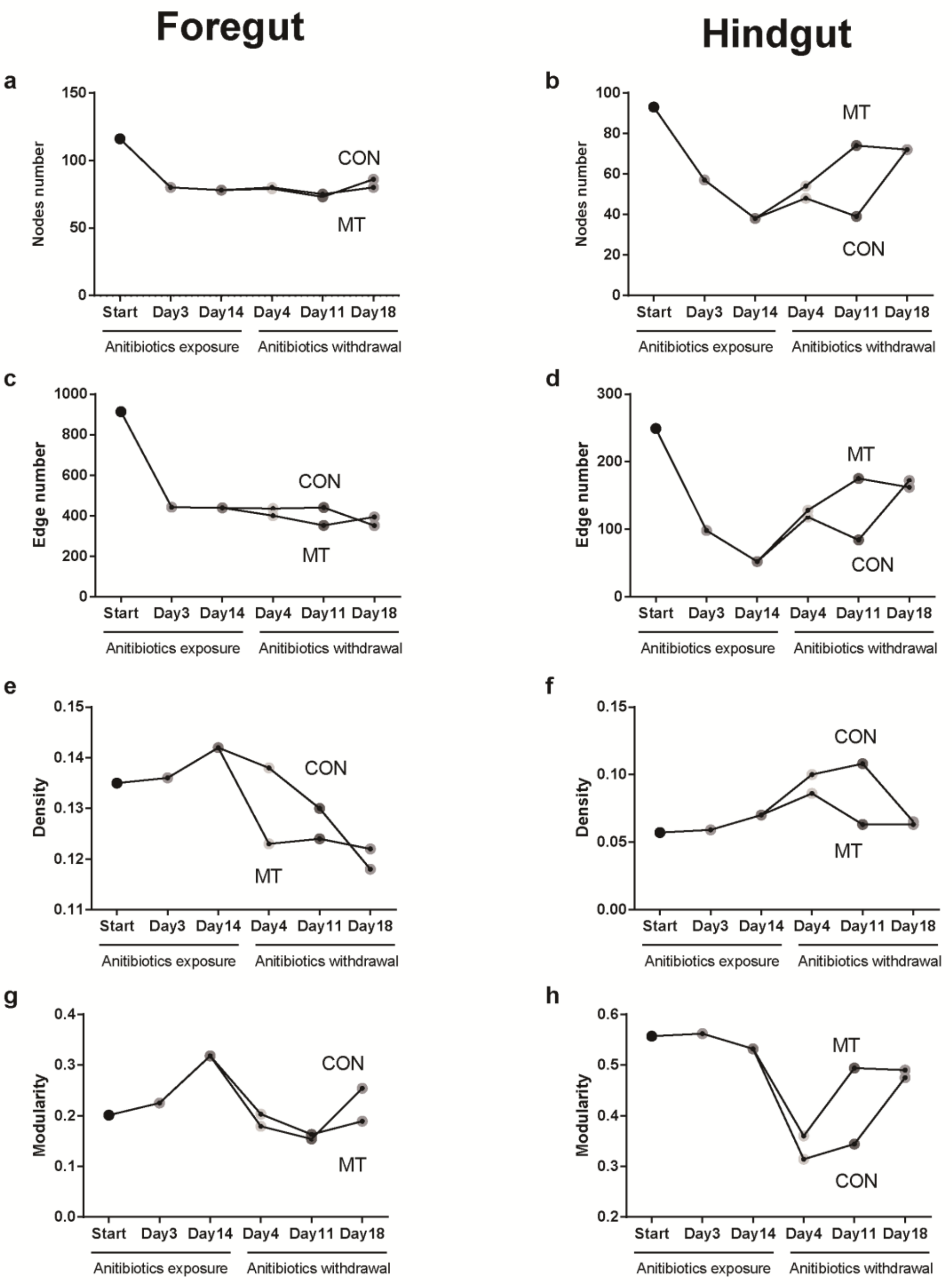

Supplementary Figure S6 Network feature change in foregut and hindgut. Nodes number (a,b), edges number (c,d), density (e,f) and modularity (g,h) change in network of microbiota.

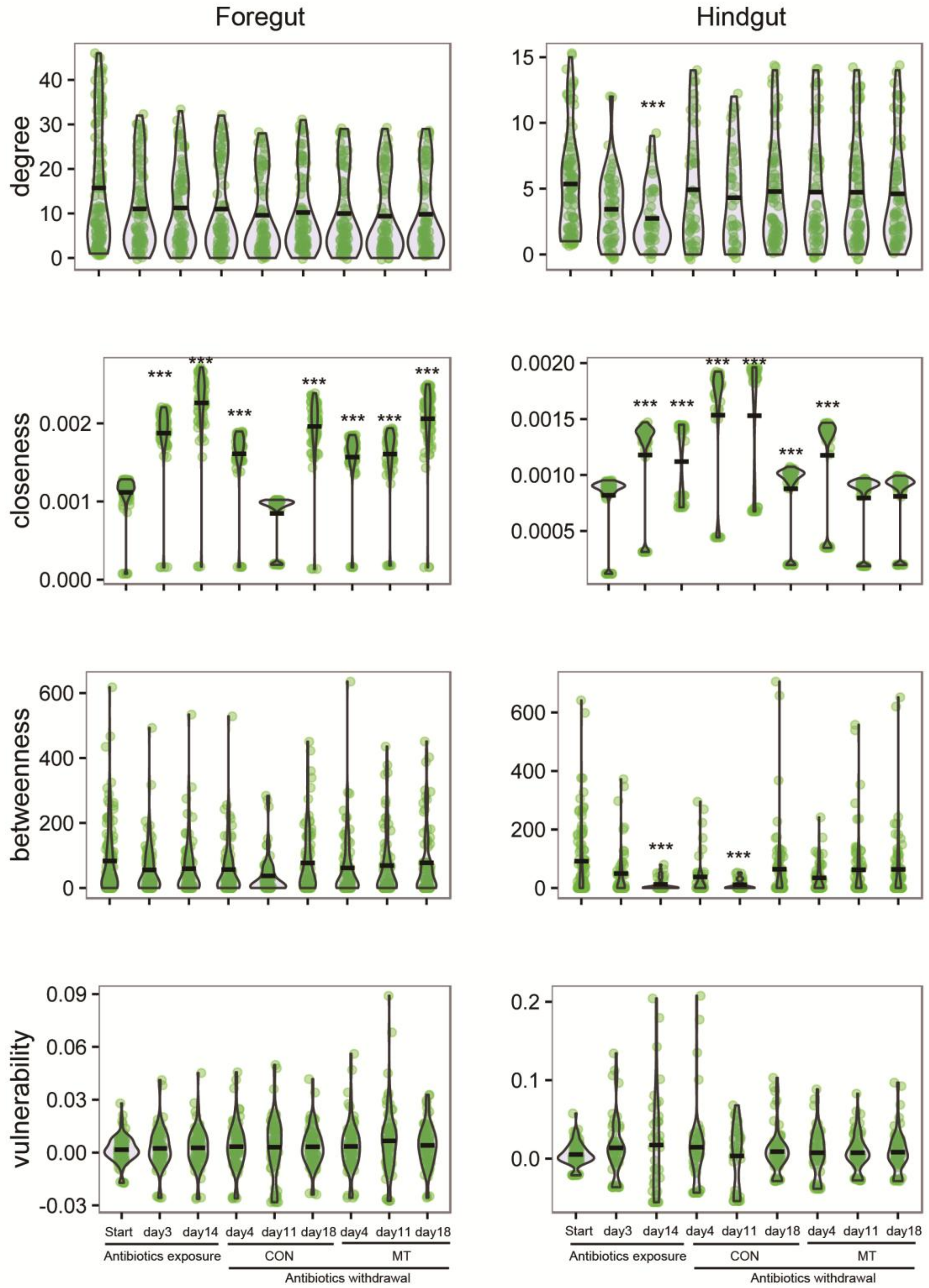

Supplementary Figure S7 Degree, closeness, betweenness and vulnerability distribution in networks of microbiota in foregut and hindgut. Violin plot was performed in R software (Version 3.3.1), means of parameters in each group were shown as black bars and significant change of each parameter comparing with start group was marked with ***$P < 0.01$.

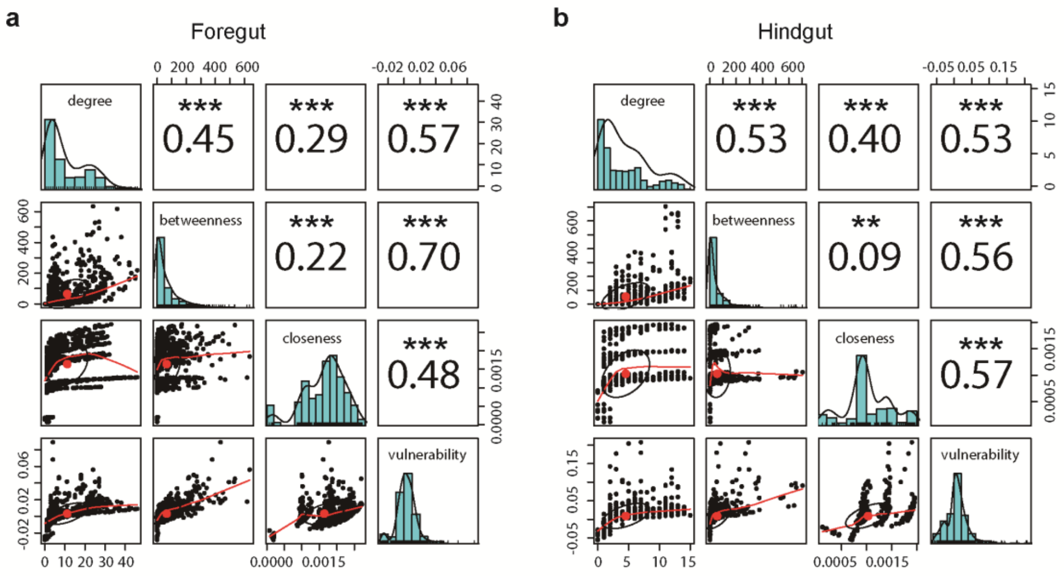

Supplementary Figure S8 Correlations of node degree, betweenness, closeness and vulnerability in network of microbiota in foregut (a) and hindgut (b), respectively. Principal diagonal represented the distributions of node degree, betweenness, closeness and vulnerability. Lower triangle panels represented the correlations of each pair parameter, while numbers in upper triangle panels represented the Pearson correlation coefficient (*r*), and significance of the correlation was marked with $**P < 0.05$, $***P < 0.01$.

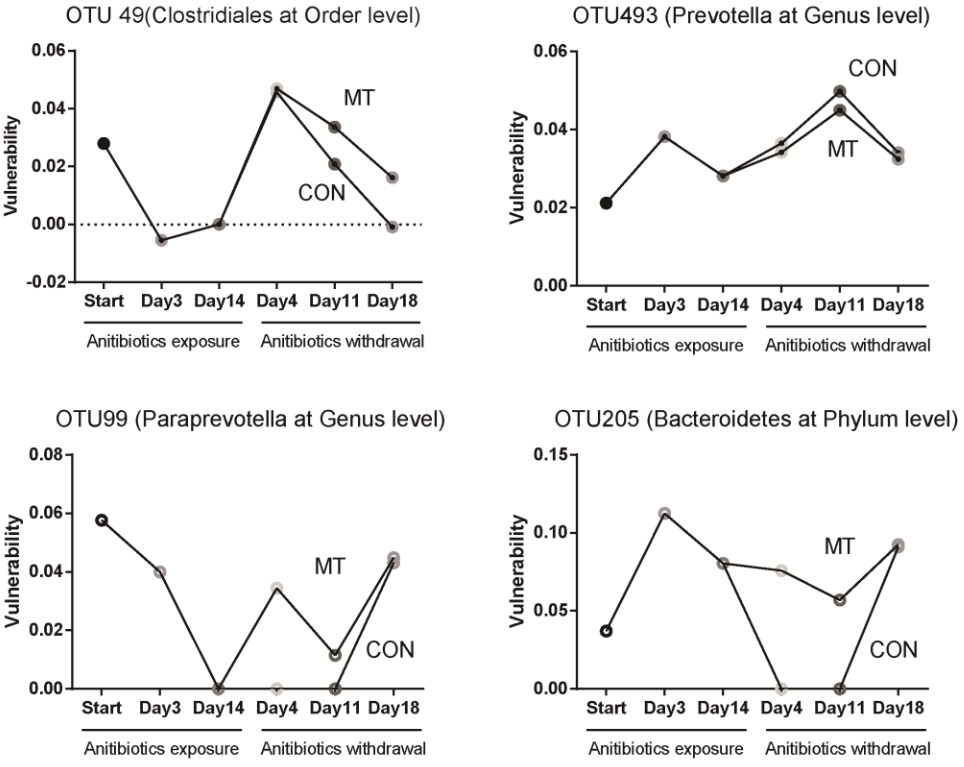

Supplementary Figure S9 Changes of the top 2 nodes with highest vulnerability in co-occurrence network in foregut (top panel) and hindgut (bottom panel).
