# Supplementary material for: Ecological Restoration of Antibiotic-Disturbed Gastrointestinal Microbiota in Foregut and Hindgut of Cows
Source: Front Cell Infect Microbiol. 2018 Mar 13;8:79. doi: 10.3389/fcimb.2018.00079 (PMC5859144; doi:10.3389/fcimb.2018.00079)
Supplement: Supplementary file 2 [file Table1.DOCX]

Supplementary Table S1 ingredients and contents as DM bases.

| Items^1^ | diet |
| --- | --- |
| Ingredients (kg) |  |
| Corn silage | 4.51 |
| Alfafa hay | 2.38 |
| Extruded soybean | 0.35 |
| Steam flaked corn | 3.26 |
| Ground corn | 1.72 |
| DDGS^1^ | 1.79 |
| Salt (Sodium chloride) | 0.08 |
| Sodium bicarbonate | 0.29 |
| Cottonseed | 1.36 |
| Soybean meal | 2.12 |
| Beet pulp | 1.00 |
| Premix | 2.04 |
| Contents (%) |  |
| DM as fed | 55.21 |
| Crude protein | 17.98 |
| NE_L_(MCal/kg)^2^ | 1.74 |
| Fat | 5.46 |
| NDF | 31.55 |
| ADF | 16.19 |
| NFC | 41.21 |
| Ca | 0.82 |
| P | 0.41 |

^1^DDGS=dried distillers grains with solubles, DM=dry matter, NE_L_=net energy requirement for lactation, NDF= neutral detergent fiber, ADF= acid detergent fiber, NFC= nonfiber carbohydrates, Ca=calcium, P=phosphorus.

^2^ Calculated using equations from NRC (2001).
