# Supplementary material for: Ecological Restoration of Antibiotic-Disturbed Gastrointestinal Microbiota in Foregut and Hindgut of Cows
Source: Front Cell Infect Microbiol. 2018 Mar 13;8:79. doi: 10.3389/fcimb.2018.00079 (PMC5859144; doi:10.3389/fcimb.2018.00079)
Supplement: Supplementary file 3 [file Table2.DOCX]

| **ID** | Taxonomy  (phylum) | Mean | | |  | SD | | |  | *P*-values | |
| --- | --- | --- | --- | --- | --- | --- | --- | --- | --- | --- | --- |
|  |  | Start | Day 3 | Day 14 |  | Start | Day 3 | Day 14 |  | Start VS Day 3 | Start VS Day 14 |
| **OTU_67** | p__Firmicutes | 0.002313 | 0.000566 | 0.000918159 |  | 0.000606898 | 0.000235983 | 0.000384619 |  | 2.17E-05 | 1.08E-05 |
| **OTU_45** | others | 0.006171 | 0.000846 | 0.00042808 |  | 0.002991699 | 0.000587208 | 0.000507863 |  | 2.17E-05 | 1.08E-05 |
| **OTU_9** | p__Firmicutes | 0.00485 | 0.00152 | 0.002315981 |  | 0.001166282 | 0.00069665 | 0.000924807 |  | 2.17E-05 | 4.33E-05 |
| **OTU_180** | p__Bacteroidetes | 0.002919 | 0.001209 | 0.001249143 |  | 0.000905123 | 0.000448354 | 0.000633029 |  | 2.17E-05 | 0.000205677 |
| **OTU_55** | p__Firmicutes | 0.010201 | 0.004162 | 0.006763136 |  | 0.003069677 | 0.001232973 | 0.002446007 |  | 2.17E-05 | 0.006841456 |
| **OTU_41** | p__Firmicutes | 0.008642 | 0.001864 | 0.006880357 |  | 0.001865374 | 0.001222288 | 0.002645972 |  | 2.17E-05 | 0.165493949 |
| **OTU_18** | p__Firmicutes | 0.00946 | 0.004316 | 0.006210191 |  | 0.00281986 | 0.001919348 | 0.002804802 |  | 8.66E-05 | 0.014689645 |
| **OTU_47** | p__Bacteroidetes | 0.003 | 0.007936 | 0.007000051 |  | 0.001294304 | 0.003261154 | 0.00311255 |  | 0.000151551 | 0.000129901 |
| **OTU_133** | p__Bacteroidetes | 0.001479 | 0.004953 | 0.00402725 |  | 0.000505191 | 0.002012834 | 0.001659958 |  | 0.000151551 | 0.000129901 |
| **OTU_128** | p__Bacteroidetes | 0.006439 | 0.002015 | 0.003901262 |  | 0.002914871 | 0.00075272 | 0.002987303 |  | 0.000151551 | 0.02880556 |
| **OTU_68** | p__Firmicutes | 0.002461 | 0.000864 | 0.000578874 |  | 0.001037382 | 0.000469249 | 0.000278072 |  | 0.000259802 | 2.17E-05 |
| **OTU_173** | p__Firmicutes | 0.002494 | 0.001139 | 0.001074565 |  | 0.000751078 | 0.000371018 | 0.000388244 |  | 0.000259802 | 7.58E-05 |
| **OTU_928** | p__Firmicutes | 0.001693 | 0.000212 | 0.001407801 |  | 0.000777766 | 0.000248324 | 0.000847615 |  | 0.000277976 | 0.853428305 |
| **OTU_48** | p__Firmicutes | 0.0036 | 0.001239 | 0.002649917 |  | 0.001200841 | 0.000869193 | 0.000619875 |  | 0.000411353 | 0.052425902 |
| **OTU_10** | p__Firmicutes | 0.010711 | 0.004817 | 0.002731748 |  | 0.003980622 | 0.001811286 | 0.001047392 |  | 0.000649505 | 1.08E-05 |
| **OTU_81** | p__Bacteroidetes | 0.00481 | 0.002106 | 0.002659443 |  | 0.001834995 | 0.000657104 | 0.001210801 |  | 0.000649505 | 0.005196042 |
| **OTU_152** | p__Firmicutes | 0.001851 | 0.004057 | 0.002850855 |  | 0.000517683 | 0.001640513 | 0.002449911 |  | 0.000649505 | 0.630528914 |
| **OTU_378** | p__Bacteroidetes | 0.00197 | 0.00102 | 0.000884434 |  | 0.000541157 | 0.000395523 | 0.000388107 |  | 0.000974258 | 0.000324753 |
| **OTU_371** | p__Firmicutes | 0.003023 | 0.001184 | 0.001735011 |  | 0.001431848 | 0.000496657 | 0.000623398 |  | 0.000974258 | 0.011496244 |
| **OTU_140** | p__Firmicutes | 0.001943 | 0.000769 | 0.001396778 |  | 0.00086366 | 0.000381317 | 0.001488167 |  | 0.000974258 | 0.02880556 |
| **OTU_304** | p__Firmicutes | 0.002799 | 0.000835 | 0.000292408 |  | 0.001280964 | 0.000430152 | 0.000221461 |  | 0.001450562 | 0.000487129 |
| **OTU_6** | p__Bacteroidetes | 0.00589 | 0.012775 | 0.012302526 |  | 0.002487743 | 0.0063864 | 0.005830753 |  | 0.001450562 | 0.001050034 |
| **OTU_126** | p__Bacteroidetes | 0.002277 | 0.001075 | 0.00194289 |  | 0.000838333 | 0.000454352 | 0.00053161 |  | 0.001450562 | 0.247450692 |
| **OTU_5** | p__Firmicutes | 0.03328 | 0.015106 | 0.033855708 |  | 0.012019822 | 0.008365603 | 0.010307816 |  | 0.001450562 | 0.97051246 |
| **OTU_8** | p__Firmicutes | 0.006073 | 0.003011 | 0.001513201 |  | 0.002374756 | 0.001030527 | 0.000825294 |  | 0.002100067 | 1.08E-05 |
| **OTU_176** | p__Firmicutes | 0.002745 | 0.000787 | 0.000387017 |  | 0.001637048 | 0.000454584 | 0.000271219 |  | 0.002100067 | 4.33E-05 |
| **OTU_398** | p__Firmicutes | 0.001719 | 0.000883 | 0.000776956 |  | 0.000574838 | 0.000459321 | 0.00025235 |  | 0.002100067 | 7.58E-05 |
| **OTU_169** | p__Firmicutes | 0.002817 | 0.001166 | 0.00079914 |  | 0.000912973 | 0.000866728 | 0.000596714 |  | 0.002987724 | 0.000205677 |
| **OTU_493** | p__Bacteroidetes | 0.002432 | 0.005039 | 0.003726055 |  | 0.001770958 | 0.001116773 | 0.00148423 |  | 0.002987724 | 0.089209552 |
| **OTU_12** | p__Bacteroidetes | 0.027054 | 0.013636 | 0.008196407 |  | 0.010048586 | 0.005368512 | 0.005133819 |  | 0.004135184 | 7.58E-05 |
| **OTU_107** | p__Firmicutes | 0.001864 | 0.00113 | 0.003691528 |  | 0.000389695 | 0.000502622 | 0.003045909 |  | 0.005672346 | 0.075256013 |
| **OTU_138** | p__Bacteroidetes | 0.001983 | 0.003684 | 0.003034104 |  | 0.001302627 | 0.001323509 | 0.001894926 |  | 0.005672346 | 0.143140142 |
| **OTU_16** | p__Firmicutes | 0.001793 | 0.001022 | 0.001482522 |  | 0.00060728 | 0.000574886 | 0.000505562 |  | 0.005672346 | 0.435872177 |
| **OTU_2** | p__Firmicutes | 0.013949 | 0.00653 | 0.007100092 |  | 0.005119752 | 0.004816173 | 0.002329048 |  | 0.007620862 | 0.000324753 |
| **OTU_14** | p__Bacteroidetes | 0.004576 | 0.01329 | 0.014696303 |  | 0.003646403 | 0.009908855 | 0.008883303 |  | 0.007620862 | 0.001050034 |
| **OTU_319** | p__Firmicutes | 0.001418 | 0.000713 | 0.000783689 |  | 0.000447981 | 0.000461979 | 0.000372722 |  | 0.007620862 | 0.003886207 |
| **OTU_1597** | p__Bacteroidetes | 0.00194 | 0.000887 | 0.001293552 |  | 0.000796467 | 0.000544578 | 0.001096469 |  | 0.007620862 | 0.089209552 |
| **OTU_23** | p__Firmicutes | 0.007884 | 0.015004 | 0.010246062 |  | 0.00323853 | 0.005692632 | 0.005312169 |  | 0.007620862 | 0.352681374 |
| **OTU_46** | p__Firmicutes | 0.002303 | 0.001007 | 0.002601114 |  | 0.001153146 | 0.000631779 | 0.001533979 |  | 0.007620862 | 0.795936262 |
| **OTU_51** | p__Firmicutes | 0.002386 | 0.001727 | 0.001333163 |  | 0.000502862 | 0.000902766 | 0.000494812 |  | 0.013271558 | 0.000487129 |
| **OTU_526** | p__Bacteroidetes | 0.005006 | 0.008666 | 0.009886254 |  | 0.002645687 | 0.002953174 | 0.003656439 |  | 0.013271558 | 0.002089242 |
| **OTU_980** | p__Bacteroidetes | 0.004321 | 0.002039 | 0.004615089 |  | 0.002233335 | 0.001348283 | 0.001409594 |  | 0.013271558 | 0.578741692 |
| **OTU_35** | p__Bacteroidetes | 0.005953 | 0.009783 | 0.010562348 |  | 0.002322545 | 0.005302385 | 0.002855169 |  | 0.01721189 | 0.000487129 |
| **OTU_139** | p__Bacteroidetes | 0.001886 | 0.003068 | 0.002594547 |  | 0.001210468 | 0.001365709 | 0.000551846 |  | 0.01721189 | 0.014689645 |
| **OTU_785** | p__Firmicutes | 0.001971 | 0.000872 | 0.001608738 |  | 0.001087265 | 0.000564113 | 0.000639567 |  | 0.01721189 | 0.739364351 |
| **OTU_54** | p__Firmicutes | 0.002642 | 0.001572 | 0.000489838 |  | 0.000995464 | 0.000601739 | 0.000149099 |  | 0.022018229 | 1.08E-05 |
| **OTU_15** | p__Firmicutes | 0.011035 | 0.006448 | 0.016831059 |  | 0.004752061 | 0.002797524 | 0.007377696 |  | 0.022018229 | 0.075256013 |
| **OTU_120** | p__Bacteroidetes | 0.004769 | 0.003002 | 0.00441474 |  | 0.002425724 | 0.001472418 | 0.001297072 |  | 0.022018229 | 0.853428305 |
| **OTU_353** | others | 0.002452 | 0.001507 | 0.000671508 |  | 0.000878568 | 0.001360817 | 0.000446147 |  | 0.027928728 | 2.17E-05 |
| **OTU_95** | p__Firmicutes | 0.003248 | 0.001886 | 0.002895463 |  | 0.000968228 | 0.001165124 | 0.00168287 |  | 0.027928728 | 0.52884886 |
| **OTU_272** | p__Bacteroidetes | 0.00267 | 0.001365 | 0.00368352 |  | 0.001511332 | 0.000657845 | 0.001676305 |  | 0.034986685 | 0.165493949 |
| **OTU_19** | p__Firmicutes | 0.016352 | 0.01193 | 0.014720567 |  | 0.004730787 | 0.004510319 | 0.007211558 |  | 0.034986685 | 0.352681374 |
| **OTU_17** | p__Firmicutes | 0.007925 | 0.005526 | 0.00417475 |  | 0.002625745 | 0.003277841 | 0.001743462 |  | 0.043473554 | 0.005196042 |
| **OTU_13** | p__Firmicutes | 0.032953 | 0.021417 | 0.021906234 |  | 0.010393958 | 0.00857954 | 0.010877884 |  | 0.043473554 | 0.043257053 |
| **OTU_161** | p__Bacteroidetes | 0.00246 | 0.001715 | 0.001754562 |  | 0.001046161 | 0.001377507 | 0.001062334 |  | 0.043473554 | 0.105122432 |
| **OTU_168** | p__Firmicutes | 0.003814 | 0.002103 | 0.003910457 |  | 0.001973517 | 0.00071987 | 0.002634117 |  | 0.043473554 | 0.911797181 |
| **OTU_201** | p__Bacteroidetes | 0.002371 | 0.001486 | 0.001120088 |  | 0.001095267 | 0.001037582 | 0.000434274 |  | 0.053475936 | 0.005196042 |
| **OTU_350** | p__Firmicutes | 0.001483 | 0.000886 | 0.00119687 |  | 0.000386308 | 0.000554717 | 0.000501456 |  | 0.053475936 | 0.105122432 |
| **OTU_711** | p__Bacteroidetes | 0.002559 | 0.00113 | 0.001488611 |  | 0.00196761 | 0.001077197 | 0.001300321 |  | 0.060276405 | 0.240613076 |
| **OTU_59** | p__Bacteroidetes | 0.001438 | 0.001872 | 0.002280435 |  | 0.000630804 | 0.000557085 | 0.000896383 |  | 0.065253632 | 0.023230639 |
| **OTU_516** | others | 0.0014 | 0.000906 | 0.00094777 |  | 0.0004796 | 0.000488484 | 0.000465 |  | 0.065253632 | 0.063012839 |
| **OTU_443** | p__Bacteroidetes | 0.001984 | 0.003336 | 0.001984637 |  | 0.000729098 | 0.001525583 | 0.001153006 |  | 0.065253632 | 0.853428305 |
| **OTU_49** | p__Firmicutes | 0.004363 | 0.002298 | 0.001106235 |  | 0.003416731 | 0.001064009 | 0.000650114 |  | 0.078893243 | 7.58E-05 |
| **OTU_188** | p__Firmicutes | 0.002673 | 0.004032 | 0.005388267 |  | 0.000720025 | 0.001398694 | 0.004762215 |  | 0.078893243 | 0.089209552 |
| **OTU_20** | p__Firmicutes | 0.005316 | 0.004133 | 0.008072147 |  | 0.002744129 | 0.004725708 | 0.004284369 |  | 0.094719522 | 0.063012839 |
| **OTU_100** | p__Firmicutes | 0.002204 | 0.001265 | 0.000840297 |  | 0.001380389 | 0.000745868 | 0.000328775 |  | 0.112754119 | 0.001504687 |
| **OTU_88** | p__Firmicutes | 0.002723 | 0.003973 | 0.004905847 |  | 0.001141101 | 0.001499744 | 0.0020749 |  | 0.112754119 | 0.002879473 |
| **OTU_234** | p__Bacteroidetes | 0.005234 | 0.002097 | 0.001568712 |  | 0.006001241 | 0.002251935 | 0.00135408 |  | 0.112754119 | 0.018543376 |
| **OTU_127** | p__Firmicutes | 0.001779 | 0.001369 | 0.001310725 |  | 0.000674714 | 0.000891652 | 0.000554231 |  | 0.133300136 | 0.105122432 |
| **OTU_52** | p__Firmicutes | 0.002528 | 0.003207 | 0.00474614 |  | 0.001601874 | 0.001401732 | 0.003545891 |  | 0.133300136 | 0.123005477 |
| **OTU_56** | others | 0.017774 | 0.012453 | 0.006217628 |  | 0.007753507 | 0.007802256 | 0.00273763 |  | 0.156400875 | 0.000205677 |
| **OTU_186** | p__Bacteroidetes | 0.005363 | 0.007407 | 0.006405278 |  | 0.002648237 | 0.003921846 | 0.001808206 |  | 0.156400875 | 0.165493949 |
| **OTU_270** | p__Bacteroidetes | 0.001677 | 0.00129 | 0.002770859 |  | 0.000627875 | 0.000676561 | 0.001445148 |  | 0.182316136 | 0.014689645 |
| **OTU_681** | p__Firmicutes | 0.001281 | 0.000913 | 0.00077786 |  | 0.000465705 | 0.000516008 | 0.000360148 |  | 0.182316136 | 0.018543376 |
| **OTU_396** | p__Bacteroidetes | 0.001756 | 0.001413 | 0.001255812 |  | 0.000553755 | 0.000580988 | 0.000497238 |  | 0.182316136 | 0.075256013 |
| **OTU_43** | p__Bacteroidetes | 0.007809 | 0.011046 | 0.008898842 |  | 0.002711317 | 0.005325095 | 0.0030475 |  | 0.182316136 | 0.481250947 |
| **OTU_289** | p__Bacteroidetes | 0.001791 | 0.001305 | 0.001949241 |  | 0.000729926 | 0.000561004 | 0.000975327 |  | 0.182316136 | 0.739364351 |
| **OTU_27** | p__Bacteroidetes | 0.009564 | 0.006567 | 0.010016386 |  | 0.005088048 | 0.003150379 | 0.006020935 |  | 0.182316136 | 0.911797181 |
| **OTU_91** | p__Firmicutes | 0.002284 | 0.002925 | 0.002321072 |  | 0.001540935 | 0.001446136 | 0.001362194 |  | 0.21102427 | 0.630528914 |
| **OTU_325** | p__Firmicutes | 0.001666 | 0.001095 | 0.001373144 |  | 0.000872103 | 0.000568981 | 0.000577733 |  | 0.21102427 | 0.911797181 |
| **OTU_707** | p__Bacteroidetes | 0.002645 | 0.001955 | 0.000903359 |  | 0.001936191 | 0.001751894 | 0.000772653 |  | 0.242806729 | 0.006841456 |
| **OTU_29** | p__Bacteroidetes | 0.009889 | 0.012579 | 0.013796014 |  | 0.010636794 | 0.009266539 | 0.008556751 |  | 0.242806729 | 0.063012839 |
| **OTU_84** | p__Bacteroidetes | 0.001589 | 0.001216 | 0.002042422 |  | 0.000729904 | 0.000787204 | 0.000730133 |  | 0.242806729 | 0.217562623 |
| **OTU_329** | p__Firmicutes | 0.001521 | 0.00274 | 0.001498828 |  | 0.000823518 | 0.00230633 | 0.001279265 |  | 0.242806729 | 0.578741692 |
| **OTU_805** | p__Firmicutes | 0.003302 | 0.004761 | 0.004701754 |  | 0.002272182 | 0.003077967 | 0.004622604 |  | 0.242806729 | 0.630528914 |
| **OTU_114** | p__Bacteroidetes | 0.002424 | 0.004825 | 0.001533348 |  | 0.001326268 | 0.003707093 | 0.00042047 |  | 0.277511962 | 0.089209552 |
| **OTU_136** | p__Bacteroidetes | 0.001802 | 0.002239 | 0.001799047 |  | 0.000904621 | 0.000913751 | 0.000689187 |  | 0.277511962 | 0.97051246 |
| **OTU_36** | p__Firmicutes | 0.003659 | 0.003116 | 0.001664309 |  | 0.000980968 | 0.001253797 | 0.000932385 |  | 0.31537812 | 0.000725281 |
| **OTU_90** | p__Firmicutes | 0.002155 | 0.001835 | 0.001120585 |  | 0.000754459 | 0.000647141 | 0.000540135 |  | 0.31537812 | 0.002879473 |
| **OTU_63** | p__Firmicutes | 0.002689 | 0.003066 | 0.00183314 |  | 0.001203631 | 0.001154319 | 0.00152449 |  | 0.31537812 | 0.014689645 |
| **OTU_25** | p__Bacteroidetes | 0.004911 | 0.003866 | 0.007746585 |  | 0.003155375 | 0.003659847 | 0.005342563 |  | 0.31537812 | 0.217562623 |
| **OTU_74** | p__Firmicutes | 0.001397 | 0.001794 | 0.00235931 |  | 0.000352792 | 0.000754393 | 0.000853003 |  | 0.356232003 | 0.005196042 |
| **OTU_97** | p__Firmicutes | 0.002842 | 0.002345 | 0.002025148 |  | 0.001237517 | 0.00070587 | 0.000969558 |  | 0.356232003 | 0.217562623 |
| **OTU_64** | others | 0.00557 | 0.009398 | 0.004367214 |  | 0.00211231 | 0.006588362 | 0.001782302 |  | 0.356232003 | 0.279861006 |
| **OTU_11** | p__Firmicutes | 0.011621 | 0.008904 | 0.008674159 |  | 0.008341428 | 0.006738341 | 0.006088046 |  | 0.356232003 | 0.314999242 |
| **OTU_103** | p__Bacteroidetes | 0.006504 | 0.004009 | 0.005521168 |  | 0.004962485 | 0.002017103 | 0.002118638 |  | 0.356232003 | 0.630528914 |
| **OTU_467** | p__Bacteroidetes | 0.004793 | 0.003196 | 0.00463335 |  | 0.003638317 | 0.002140554 | 0.004399447 |  | 0.356232003 | 0.684210526 |
| **OTU_50** | p__Bacteroidetes | 0.005154 | 0.004367 | 0.005077161 |  | 0.001869663 | 0.001428 | 0.001748901 |  | 0.356232003 | 0.853428305 |
| **OTU_42** | p__Bacteroidetes | 0.006768 | 0.003421 | 0.010998443 |  | 0.008389719 | 0.001294878 | 0.011296242 |  | 0.400181861 | 0.247450692 |
| **OTU_44** | p__Bacteroidetes | 0.006696 | 0.008842 | 0.007924832 |  | 0.002876535 | 0.004469144 | 0.002768944 |  | 0.400181861 | 0.352681374 |
| **OTU_38** | p__Firmicutes | 0.001666 | 0.002211 | 0.002072286 |  | 0.000585079 | 0.001055302 | 0.001509629 |  | 0.400181861 | 0.853428305 |
| **OTU_220** | p__Bacteroidetes | 0.001393 | 0.001369 | 0.000525981 |  | 0.000515899 | 0.001046519 | 0.000208943 |  | 0.446967893 | 0.000205677 |
| **OTU_1106** | p__Bacteroidetes | 0.00205 | 0.002505 | 0.002513523 |  | 0.001039084 | 0.001320425 | 0.000683053 |  | 0.446967893 | 0.190315876 |
| **OTU_174** | others | 0.001623 | 0.001324 | 0.001251356 |  | 0.000774103 | 0.000682549 | 0.000670472 |  | 0.446967893 | 0.314999242 |
| **OTU_30** | p__Bacteroidetes | 0.021731 | 0.017848 | 0.02111776 |  | 0.008545793 | 0.006738906 | 0.007136143 |  | 0.446967893 | 0.911797181 |
| **OTU_117** | p__Bacteroidetes | 0.004374 | 0.002753 | 0.003457807 |  | 0.003900513 | 0.002480108 | 0.001631596 |  | 0.446967893 | 0.97051246 |
| **OTU_73** | p__Bacteroidetes | 0.001568 | 0.001953 | 0.002309289 |  | 0.000545154 | 0.000953129 | 0.0009478 |  | 0.496698348 | 0.02880556 |
| **OTU_53** | p__Bacteroidetes | 0.00781 | 0.009798 | 0.011022271 |  | 0.003544359 | 0.005242566 | 0.003320498 |  | 0.496698348 | 0.043257053 |
| **OTU_1660** | p__Bacteroidetes | 0.001724 | 0.001693 | 0.001651743 |  | 0.000770928 | 0.00129076 | 0.000612318 |  | 0.496698348 | 0.911797181 |
| **OTU_34** | p__Firmicutes | 0.002365 | 0.001966 | 0.001326105 |  | 0.001519423 | 0.001992502 | 0.001081961 |  | 0.548961874 | 0.123005477 |
| **OTU_72** | p__Firmicutes | 0.004526 | 0.003254 | 0.003065024 |  | 0.003644932 | 0.002314709 | 0.00197563 |  | 0.548961874 | 0.630528914 |
| **OTU_87** | others | 0.009561 | 0.01052 | 0.006408264 |  | 0.004169975 | 0.005018389 | 0.005072072 |  | 0.603780121 | 0.018543376 |
| **OTU_24** | p__Bacteroidetes | 0.006068 | 0.006588 | 0.010774273 |  | 0.004212654 | 0.00434231 | 0.004750538 |  | 0.603780121 | 0.035462989 |
| **OTU_547** | p__Firmicutes | 0.00155 | 0.001966 | 0.001350425 |  | 0.000589314 | 0.001065206 | 0.000870044 |  | 0.603780121 | 0.314999242 |
| **OTU_28** | p__Bacteroidetes | 0.004303 | 0.006063 | 0.004059531 |  | 0.001580257 | 0.005177866 | 0.001458924 |  | 0.603780121 | 0.911797181 |
| **OTU_106** | others | 0.002746 | 0.004707 | 0.001761664 |  | 0.001041701 | 0.00456408 | 0.001438309 |  | 0.660720085 | 0.023230639 |
| **OTU_215** | p__Firmicutes | 0.002393 | 0.004019 | 0.003108761 |  | 0.001802772 | 0.004495728 | 0.001911828 |  | 0.660720085 | 0.352681374 |
| **OTU_273** | others | 0.001625 | 0.001936 | 0.00112207 |  | 0.000730151 | 0.001043198 | 0.000362641 |  | 0.719695166 | 0.105122432 |
| **OTU_643** | p__Bacteroidetes | 0.002816 | 0.003827 | 0.003028686 |  | 0.001708374 | 0.003024904 | 0.001959649 |  | 0.719695166 | 0.739364351 |
| **OTU_116** | p__Bacteroidetes | 0.004408 | 0.005687 | 0.002576115 |  | 0.002999722 | 0.004651684 | 0.000978699 |  | 0.780185759 | 0.165493949 |
| **OTU_98** | p__Bacteroidetes | 0.003816 | 0.003452 | 0.003658723 |  | 0.002303498 | 0.00220994 | 0.002258748 |  | 0.780185759 | 0.97051246 |
| **OTU_214** | p__Bacteroidetes | 0.001456 | 0.00134 | 0.000420275 |  | 0.000436086 | 0.000685035 | 0.000227015 |  | 0.842105263 | 1.08E-05 |
| **OTU_536** | p__Bacteroidetes | 0.001346 | 0.001608 | 0.001337629 |  | 0.000351442 | 0.000651325 | 0.00040163 |  | 0.842105263 | 0.97051246 |
| **OTU_151** | p__Bacteroidetes | 0.002141 | 0.002351 | 0.00314629 |  | 0.001016942 | 0.001156909 | 0.001303729 |  | 0.968239191 | 0.089209552 |
| **OTU_208** | p__Bacteroidetes | 0.0028 | 0.002206 | 0.003135317 |  | 0.002049312 | 0.001050384 | 0.002978897 |  | 1 | 0.853428305 |
| **OTU_229** | others | 0.002956 | 0.002906 | 0.003200624 |  | 0.001266515 | 0.001370514 | 0.002036994 |  | 1 | 0.911797181 |
