# Supplementary material for: Ecological Restoration of Antibiotic-Disturbed Gastrointestinal Microbiota in Foregut and Hindgut of Cows
Source: Front Cell Infect Microbiol. 2018 Mar 13;8:79. doi: 10.3389/fcimb.2018.00079 (PMC5859144; doi:10.3389/fcimb.2018.00079)
Supplement: Supplementary file 4 [file Table3.DOCX]

| **ID** | taxonomy  (Phylum) | Mean | | |  | SD | | |  | *P*-values | |
| --- | --- | --- | --- | --- | --- | --- | --- | --- | --- | --- | --- |
|  |  | Start | Day 3 | Day 14 |  | Start | Day 3 | Day 14 |  | Start VS Day 3 | Start VS Day 14 |
| **OTU_2** | p__Firmicutes | 0.006234 | 0.000732 | 0.000386 |  | 0.002622 | 0.000615 | 0.000718 |  | 1.08E-05 | 0.000178614 |
| **OTU_153** | p__Firmicutes | 0.002973 | 0.000195 | 0.000486 |  | 0.001344 | 0.000266 | 0.000766 |  | 1.08E-05 | 0.001003556 |
| **OTU_21** | p__Firmicutes | 0.032323 | 0.009706 | 0.032544 |  | 0.009718 | 0.003266 | 0.016245 |  | 1.08E-05 | 0.630528914 |
| **OTU_12** | p__Firmicutes | 0.005152 | 0.000138 | 0.00837 |  | 0.003405 | 0.000117 | 0.007975 |  | 1.08E-05 | 0.739364351 |
| **OTU_144** | p__Firmicutes | 0.010415 | 0.004091 | 0.00231 |  | 0.00316 | 0.002189 | 0.001585 |  | 4.33E-05 | 1.08E-05 |
| **OTU_123** | p__Firmicutes | 0.002716 | 0.000739 | 0.000775 |  | 0.000922 | 0.000334 | 0.000543 |  | 4.33E-05 | 0.000129901 |
| **OTU_142** | p__Firmicutes | 0.002302 | 0 | 0.000261 |  | 0.00157 | 0 | 0.000356 |  | 6.39E-05 | 0.000163049 |
| **OTU_93** | p__Firmicutes | 0.004422 | 0.001408 | 0.000951 |  | 0.001252 | 0.000894 | 0.000429 |  | 7.58E-05 | 1.08E-05 |
| **OTU_180** | p__Firmicutes | 0.002366 | 4.34E-06 | 0.000846 |  | 0.000829 | 1.37E-05 | 0.000862 |  | 8.74E-05 | 0.001504687 |
| **OTU_79** | p__Firmicutes | 0.002042 | 6.34E-05 | 0.002081 |  | 0.000898 | 0.000108 | 0.001892 |  | 0.00013173 | 0.909688076 |
| **OTU_161** | p__Firmicutes | 0.005762 | 1.24E-05 | 3.02E-06 |  | 0.004004 | 1.62E-05 | 9.55E-06 |  | 0.000149393 | 8.74E-05 |
| **OTU_405** | p__Firmicutes | 0.001103 | 4.12E-05 | 0.000153 |  | 0.00014 | 5.36E-05 | 0.000209 |  | 0.000163049 | 0.000172654 |
| **OTU_174** | p__Firmicutes | 0.001658 | 0.000436 | 0.000474 |  | 0.000457 | 0.000336 | 0.000844 |  | 0.000181651 | 0.001504687 |
| **OTU_721** | p__Bacteroidetes | 0.00355 | 0.007011 | 0.002167 |  | 0.001292 | 0.001841 | 0.001109 |  | 0.000205677 | 0.02880556 |
| **OTU_5** | p__Firmicutes | 0.157147 | 0.092176 | 0.166732 |  | 0.032188 | 0.02883 | 0.040416 |  | 0.000205677 | 0.630528914 |
| **OTU_17** | p__Firmicutes | 0.002641 | 0.000138 | 0.000179 |  | 0.00144 | 0.000249 | 0.00033 |  | 0.000233127 | 0.000179701 |
| **OTU_10** | p__Firmicutes | 0.009231 | 0.003614 | 0.00063 |  | 0.002481 | 0.002543 | 0.000821 |  | 0.000324753 | 1.08E-05 |
| **OTU_257** | p__Firmicutes | 0.001748 | 0.000585 | 0.003014 |  | 0.000721 | 0.000375 | 0.002117 |  | 0.000487129 | 0.190315876 |
| **OTU_151** | p__Firmicutes | 0.002825 | 0.00068 | 0.000495 |  | 0.000753 | 0.000602 | 0.000385 |  | 0.00058006 | 2.17E-05 |
| **OTU_87** | others | 0.006096 | 0.001877 | 0.002329 |  | 0.001461 | 0.002038 | 0.001753 |  | 0.000725281 | 0.000205677 |
| **OTU_105** | p__Firmicutes | 0.007014 | 0.004083 | 0.004243 |  | 0.001553 | 0.001583 | 0.003357 |  | 0.000725281 | 0.075256013 |
| **OTU_148** | p__Firmicutes | 0.002274 | 0.000273 | 4.78E-05 |  | 0.001847 | 0.000715 | 8.52E-05 |  | 0.001476741 | 0.000149393 |
| **OTU_6** | p__Firmicutes | 0.00866 | 0.003452 | 0.001789 |  | 0.00274 | 0.002817 | 0.002898 |  | 0.001504687 | 0.000205677 |
| **OTU_266** | p__Firmicutes | 0.011044 | 0.005913 | 0.008345 |  | 0.003675 | 0.002656 | 0.005575 |  | 0.001504687 | 0.123005477 |
| **OTU_107** | p__Firmicutes | 0.001751 | 0.000397 | 0.000155 |  | 0.000879 | 0.000586 | 0.000231 |  | 0.001678936 | 0.000172654 |
| **OTU_192** | p__Firmicutes | 0.001988 | 0.000619 | 0.000222 |  | 0.000758 | 0.000647 | 0.00033 |  | 0.001699395 | 0.000163049 |
| **OTU_45** | others | 0.002888 | 0.000664 | 0.003849 |  | 0.00219 | 0.002074 | 0.004082 |  | 0.001759555 | 0.684210526 |
| **OTU_54** | p__Bacteroidetes | 0.013637 | 0.008669 | 0.009593 |  | 0.003559 | 0.008227 | 0.00367 |  | 0.002089242 | 0.023230639 |
| **OTU_690** | p__Firmicutes | 0.002754 | 0.000909 | 0.001748 |  | 0.001483 | 0.000982 | 0.000752 |  | 0.002089242 | 0.089209552 |
| **OTU_81** | p__Firmicutes | 0.001715 | 0.000467 | 0.002719 |  | 0.000885 | 0.001146 | 0.002722 |  | 0.002624317 | 0.97051246 |
| **OTU_75** | p__Firmicutes | 0.01545 | 0.008126 | 0.003738 |  | 0.004251 | 0.005153 | 0.001925 |  | 0.002879473 | 1.08E-05 |
| **OTU_559** | p__Bacteroidetes | 0.005251 | 0.000926 | 0.000697 |  | 0.005352 | 0.001577 | 0.001113 |  | 0.00356016 | 0.005612996 |
| **OTU_175** | p__Firmicutes | 0.002494 | 0.001348 | 0.00044 |  | 0.000864 | 0.000979 | 0.000358 |  | 0.003886207 | 1.08E-05 |
| **OTU_39** | p__Firmicutes | 0.006743 | 0.003856 | 0.001125 |  | 0.001663 | 0.00208 | 0.000959 |  | 0.003886207 | 2.17E-05 |
| **OTU_117** | p__Firmicutes | 0.002967 | 0.001878 | 0.000885 |  | 0.000945 | 0.000717 | 0.000986 |  | 0.003886207 | 0.000487129 |
| **OTU_4** | p__Firmicutes | 0.01294 | 0.027093 | 0.050111 |  | 0.004609 | 0.011105 | 0.053269 |  | 0.003886207 | 0.001504687 |
| **OTU_634** | p__Firmicutes | 0.001396 | 0.00073 | 0.00109 |  | 0.000434 | 0.000465 | 0.000403 |  | 0.003886207 | 0.123005477 |
| **OTU_904** | p__Bacteroidetes | 0.002855 | 0.000866 | 0.001808 |  | 0.001494 | 0.000681 | 0.001467 |  | 0.005196042 | 0.089209552 |
| **OTU_36** | p__Firmicutes | 0.006898 | 0.011859 | 0.009306 |  | 0.002486 | 0.004805 | 0.003343 |  | 0.005196042 | 0.123005477 |
| **OTU_15** | p__Bacteroidetes | 0.008814 | 0.027114 | 0.056495 |  | 0.007072 | 0.019296 | 0.025115 |  | 0.006841456 | 2.17E-05 |
| **OTU_120** | p__Firmicutes | 0.002762 | 0.001303 | 0.000922 |  | 0.001697 | 0.001955 | 0.001237 |  | 0.006841456 | 0.005776979 |
| **OTU_3** | p__Bacteroidetes | 0.010171 | 0.051345 | 0.008694 |  | 0.005142 | 0.050197 | 0.006072 |  | 0.006841456 | 0.435872177 |
| **OTU_211** | p__Firmicutes | 0.001852 | 0.000944 | 0.000271 |  | 0.000765 | 0.000658 | 0.000406 |  | 0.011496244 | 7.58E-05 |
| **OTU_295** | p__Firmicutes | 0.004762 | 0.001886 | 0.00405 |  | 0.002881 | 0.001548 | 0.004085 |  | 0.011496244 | 0.217562623 |
| **OTU_425** | p__Firmicutes | 0.007599 | 0.018133 | 0.01258 |  | 0.006525 | 0.010205 | 0.015445 |  | 0.011496244 | 0.853428305 |
| **OTU_300** | p__Firmicutes | 0.001792 | 0.000784 | 0.001377 |  | 0.000616 | 0.000868 | 0.000856 |  | 0.014689645 | 0.089209552 |
| **OTU_24** | p__Firmicutes | 0.007896 | 0.004074 | 0.005652 |  | 0.003989 | 0.003427 | 0.004347 |  | 0.018543376 | 0.105122432 |
| **OTU_38** | p__Firmicutes | 0.002104 | 0.00075 | 0.003901 |  | 0.001829 | 0.000977 | 0.006131 |  | 0.018543376 | 0.578741692 |
| **OTU_154** | p__Bacteroidetes | 0.00663 | 0.011619 | 0.003548 |  | 0.003581 | 0.004905 | 0.001632 |  | 0.023230639 | 0.023230639 |
| **OTU_76** | p__Firmicutes | 0.002615 | 0.001161 | 0.000532 |  | 0.001512 | 0.000711 | 0.000562 |  | 0.02880556 | 0.000487129 |
| **OTU_27** | p__Bacteroidetes | 0.009483 | 0.004534 | 0.00285 |  | 0.006388 | 0.005226 | 0.002834 |  | 0.02880556 | 0.000725281 |
| **OTU_26** | p__Firmicutes | 0.001453 | 0.000778 | 0.000438 |  | 0.000556 | 0.000644 | 0.00084 |  | 0.035462989 | 0.008849481 |
| **OTU_631** | p__Firmicutes | 0.00252 | 0.006346 | 0.001569 |  | 0.000972 | 0.00545 | 0.000948 |  | 0.043257053 | 0.063012839 |
| **OTU_58** | p__Firmicutes | 0.002546 | 0.001586 | 0.003289 |  | 0.001072 | 0.00077 | 0.001498 |  | 0.043257053 | 0.279861006 |
| **OTU_157** | p__Firmicutes | 0.001845 | 0.001244 | 0.00031 |  | 0.001225 | 0.002011 | 0.000365 |  | 0.045073786 | 0.000129901 |
| **OTU_110** | p__Firmicutes | 0.001696 | 0.001172 | 0.000169 |  | 0.000591 | 0.000746 | 0.000197 |  | 0.063012839 | 0.000328133 |
| **OTU_18** | p__Firmicutes | 0.001881 | 0.003247 | 0.006167 |  | 0.000965 | 0.001708 | 0.003512 |  | 0.063012839 | 0.000725281 |
| **OTU_50** | p__Firmicutes | 0.001647 | 0.001072 | 0.00106 |  | 0.000516 | 0.00058 | 0.001217 |  | 0.063012839 | 0.02880556 |
| **OTU_119** | p__Firmicutes | 0.002502 | 0.00389 | 0.003669 |  | 0.000958 | 0.002081 | 0.002366 |  | 0.063012839 | 0.217562623 |
| **OTU_135** | p__Bacteroidetes | 0.004469 | 0.003151 | 0.002067 |  | 0.002251 | 0.001919 | 0.000924 |  | 0.075256013 | 0.002089242 |
| **OTU_127** | p__Firmicutes | 0.002387 | 0.001417 | 0.000627 |  | 0.001195 | 0.001081 | 0.001021 |  | 0.075256013 | 0.007196921 |
| **OTU_16** | p__Firmicutes | 0.016221 | 0.035296 | 0.013495 |  | 0.004243 | 0.026113 | 0.009572 |  | 0.075256013 | 0.247450692 |
| **OTU_1267** | p__Firmicutes | 0.004716 | 0.003104 | 0.003363 |  | 0.001938 | 0.002261 | 0.003501 |  | 0.089209552 | 0.075256013 |
| **OTU_152** | p__Bacteroidetes | 0.0099 | 0.015991 | 0.005976 |  | 0.003563 | 0.009717 | 0.002653 |  | 0.105122432 | 0.018543376 |
| **OTU_47** | p__Firmicutes | 0.005601 | 0.003974 | 0.000655 |  | 0.001884 | 0.00259 | 0.000975 |  | 0.123005477 | 1.08E-05 |
| **OTU_630** | p__Firmicutes | 0.001583 | 0.00291 | 0.000966 |  | 0.000862 | 0.001792 | 0.000795 |  | 0.123005477 | 0.165493949 |
| **OTU_137** | p__Bacteroidetes | 0.002387 | 0.002188 | 0.00194 |  | 0.000916 | 0.002584 | 0.001244 |  | 0.123005477 | 0.217562623 |
| **OTU_495** | p__Firmicutes | 0.001425 | 0.002638 | 0.001485 |  | 0.000583 | 0.002313 | 0.001183 |  | 0.123005477 | 0.435872177 |
| **OTU_283** | p__Bacteroidetes | 0.003835 | 0.007673 | 0.003985 |  | 0.001507 | 0.007202 | 0.001984 |  | 0.123005477 | 0.795936262 |
| **OTU_145** | p__Bacteroidetes | 0.001849 | 0.001471 | 0.001097 |  | 0.001001 | 0.002201 | 0.001293 |  | 0.139867911 | 0.088492571 |
| **OTU_33** | p__Firmicutes | 0.003585 | 0.007841 | 0.008562 |  | 0.001791 | 0.006199 | 0.01081 |  | 0.143140142 | 0.143140142 |
| **OTU_130** | p__Firmicutes | 0.003125 | 0.002493 | 0.002217 |  | 0.001789 | 0.003352 | 0.002223 |  | 0.143140142 | 0.165493949 |
| **OTU_217** | p__Bacteroidetes | 0.004054 | 0.003031 | 0.003882 |  | 0.00113 | 0.001813 | 0.002591 |  | 0.143140142 | 0.352681374 |
| **OTU_52** | p__Firmicutes | 0.003328 | 0.005378 | 0.003431 |  | 0.001683 | 0.002767 | 0.002965 |  | 0.143140142 | 0.739364351 |
| **OTU_84** | p__Firmicutes | 0.002491 | 0.004071 | 0.00169 |  | 0.001487 | 0.002294 | 0.000769 |  | 0.165493949 | 0.247450692 |
| **OTU_31** | p__Bacteroidetes | 0.002859 | 0.00541 | 0.005296 |  | 0.002014 | 0.00406 | 0.003304 |  | 0.190315876 | 0.043257053 |
| **OTU_55** | p__Firmicutes | 0.013266 | 0.009618 | 0.021196 |  | 0.008845 | 0.010783 | 0.023 |  | 0.190315876 | 0.578741692 |
| **OTU_342** | p__Bacteroidetes | 0.001945 | 0.001835 | 0.0016 |  | 0.000512 | 0.001075 | 0.001048 |  | 0.217562623 | 0.352681374 |
| **OTU_205** | p__Bacteroidetes | 0.009219 | 0.008884 | 0.004768 |  | 0.001133 | 0.004356 | 0.000947 |  | 0.247450692 | 1.08E-05 |
| **OTU_67** | p__Bacteroidetes | 0.015772 | 0.022232 | 0.007243 |  | 0.006751 | 0.013665 | 0.001946 |  | 0.314999242 | 0.000205677 |
| **OTU_41** | p__Firmicutes | 0.004045 | 0.00362 | 0.004359 |  | 0.001328 | 0.001517 | 0.002476 |  | 0.314999242 | 0.52884886 |
| **OTU_982** | p__Firmicutes | 0.00833 | 0.006907 | 0.008807 |  | 0.005604 | 0.005569 | 0.008456 |  | 0.314999242 | 0.853428305 |
| **OTU_930** | p__Bacteroidetes | 0.004953 | 0.007016 | 0.002672 |  | 0.001848 | 0.003983 | 0.0009 |  | 0.352681374 | 0.001050034 |
| **OTU_159** | p__Firmicutes | 0.001627 | 0.001651 | 0.002052 |  | 0.000855 | 0.001679 | 0.001668 |  | 0.352681374 | 0.911797181 |
| **OTU_125** | p__Firmicutes | 0.001649 | 0.002914 | 0.001732 |  | 0.001054 | 0.003096 | 0.001608 |  | 0.352681374 | 0.97051246 |
| **OTU_59** | p__Firmicutes | 0.002037 | 0.002899 | 0.007063 |  | 0.001447 | 0.002123 | 0.005507 |  | 0.393048128 | 0.000324753 |
| **OTU_435** | p__Bacteroidetes | 0.002346 | 0.002201 | 0.001544 |  | 0.001563 | 0.001862 | 0.001071 |  | 0.393048128 | 0.089209552 |
| **OTU_132** | p__Firmicutes | 0.002148 | 0.002097 | 0.000401 |  | 0.000725 | 0.001469 | 0.000498 |  | 0.435872177 | 7.58E-05 |
| **OTU_852** | p__Bacteroidetes | 0.001422 | 0.001826 | 0.001407 |  | 0.000405 | 0.000846 | 0.0013 |  | 0.435872177 | 0.247450692 |
| **OTU_138** | p__Firmicutes | 0.001866 | 0.002388 | 0.001581 |  | 0.000609 | 0.001397 | 0.001143 |  | 0.435872177 | 0.481250947 |
| **OTU_20** | p__Firmicutes | 0.005392 | 0.005193 | 0.003729 |  | 0.001879 | 0.002945 | 0.001961 |  | 0.481250947 | 0.035462989 |
| **OTU_355** | p__Firmicutes | 0.003023 | 0.002525 | 0.006064 |  | 0.001726 | 0.002606 | 0.005477 |  | 0.481250947 | 0.314999242 |
| **OTU_102** | p__Bacteroidetes | 0.003818 | 0.005116 | 0.003008 |  | 0.001961 | 0.00329 | 0.001831 |  | 0.481250947 | 0.393048128 |
| **OTU_265** | p__Bacteroidetes | 0.003201 | 0.002952 | 0.001911 |  | 0.001696 | 0.001885 | 0.001525 |  | 0.52884886 | 0.052425902 |
| **OTU_530** | p__Bacteroidetes | 0.001864 | 0.001425 | 0.001273 |  | 0.001353 | 0.001081 | 0.001453 |  | 0.52884886 | 0.217562623 |
| **OTU_35** | p__Firmicutes | 0.001818 | 0.002053 | 0.001217 |  | 0.000616 | 0.000822 | 0.000687 |  | 0.578741692 | 0.035462989 |
| **OTU_44** | p__Firmicutes | 0.00673 | 0.007555 | 0.00699 |  | 0.002091 | 0.002991 | 0.004017 |  | 0.578741692 | 1 |
| **OTU_88** | p__Firmicutes | 0.002446 | 0.002078 | 0.002322 |  | 0.000926 | 0.001085 | 0.001088 |  | 0.578741692 | 1 |
| **OTU_113** | p__Firmicutes | 0.006016 | 0.007525 | 0.003578 |  | 0.004043 | 0.004888 | 0.004412 |  | 0.630528914 | 0.035462989 |
| **OTU_62** | p__Bacteroidetes | 0.00454 | 0.007657 | 0.002799 |  | 0.00304 | 0.010882 | 0.004769 |  | 0.677126446 | 0.103589204 |
| **OTU_57** | p__Firmicutes | 0.00391 | 0.004027 | 0.00041 |  | 0.001049 | 0.003307 | 0.000742 |  | 0.684210526 | 0.000296925 |
| **OTU_267** | p__Bacteroidetes | 0.00258 | 0.002378 | 0.002015 |  | 0.001078 | 0.001772 | 0.002252 |  | 0.684210526 | 0.123005477 |
| **OTU_99** | p__Bacteroidetes | 0.011421 | 0.01043 | 0.001821 |  | 0.004584 | 0.005899 | 0.001589 |  | 0.739364351 | 1.08E-05 |
| **OTU_29** | p__Firmicutes | 0.004259 | 0.004906 | 0.001111 |  | 0.001414 | 0.003905 | 0.001492 |  | 0.739364351 | 0.001003556 |
| **OTU_244** | p__Firmicutes | 0.001923 | 0.002515 | 0.000789 |  | 0.00081 | 0.001933 | 0.00063 |  | 0.739364351 | 0.002089242 |
| **OTU_56** | p__Firmicutes | 0.004559 | 0.005746 | 0.003902 |  | 0.001216 | 0.003336 | 0.002832 |  | 0.739364351 | 0.165493949 |
| **OTU_43** | p__Firmicutes | 0.00498 | 0.005021 | 0.004001 |  | 0.002091 | 0.002638 | 0.002647 |  | 0.739364351 | 0.314999242 |
| **OTU_199** | p__Bacteroidetes | 0.0045 | 0.004978 | 0.004623 |  | 0.002368 | 0.004415 | 0.00201 |  | 0.739364351 | 0.630528914 |
| **OTU_870** | p__Bacteroidetes | 0.004994 | 0.010263 | 0.005776 |  | 0.001418 | 0.012469 | 0.003451 |  | 0.739364351 | 0.97051246 |
| **OTU_232** | p__Bacteroidetes | 0.003355 | 0.005619 | 0.002049 |  | 0.001277 | 0.008309 | 0.001164 |  | 0.795936262 | 0.052425902 |
| **OTU_71** | p__Firmicutes | 0.001831 | 0.00217 | 0.001783 |  | 0.000658 | 0.00172 | 0.001623 |  | 0.795936262 | 0.435872177 |
| **OTU_22** | p__Firmicutes | 0.009904 | 0.009788 | 0.010932 |  | 0.00404 | 0.006477 | 0.00644 |  | 0.795936262 | 1 |
| **OTU_109** | p__Bacteroidetes | 0.003538 | 0.005132 | 0.00172 |  | 0.002206 | 0.005923 | 0.001329 |  | 0.853428305 | 0.014689645 |
| **OTU_53** | p__Firmicutes | 0.0037 | 0.005247 | 0.001969 |  | 0.002013 | 0.00616 | 0.00195 |  | 0.853428305 | 0.052425902 |
| **OTU_139** | p__Firmicutes | 0.003292 | 0.003289 | 0.000825 |  | 0.000936 | 0.001648 | 0.000698 |  | 0.911797181 | 2.17E-05 |
| **OTU_85** | p__Bacteroidetes | 0.004878 | 0.005164 | 0.002196 |  | 0.002115 | 0.003108 | 0.001811 |  | 0.911797181 | 0.011496244 |
| **OTU_356** | p__Firmicutes | 0.002226 | 0.002673 | 0.000973 |  | 0.001058 | 0.002393 | 0.000816 |  | 0.911797181 | 0.014689645 |
| **OTU_233** | p__Firmicutes | 0.004576 | 0.003871 | 0.006881 |  | 0.002993 | 0.002827 | 0.006109 |  | 0.911797181 | 0.314999242 |
| **OTU_299** | p__Bacteroidetes | 0.002235 | 0.002308 | 0.001385 |  | 0.001253 | 0.001333 | 0.000965 |  | 0.97051246 | 0.123005477 |
| **OTU_14** | p__Firmicutes | 0.005717 | 0.006355 | 0.01481 |  | 0.00339 | 0.004161 | 0.011262 |  | 1 | 0.023230639 |
